# Immunogenomic pan-cancer landscape reveals immune escape mechanisms and immunoediting histories

**DOI:** 10.1038/s41598-021-95287-x

**Published:** 2021-08-03

**Authors:** Shinichi Mizuno, Rui Yamaguchi, Takanori Hasegawa, Shuto Hayashi, Masashi Fujita, Fan Zhang, Youngil Koh, Su-Yeon Lee, Sung-Soo Yoon, Eigo Shimizu, Mitsuhiro Komura, Akihiro Fujimoto, Momoko Nagai, Mamoru Kato, Han Liang, Satoru Miyano, Zemin Zhang, Hidewaki Nakagawa, Seiya Imoto

**Affiliations:** 1 Center for Advanced Medical Innovation, Kyushu University, Fukuoka, Japan; 2 Human Genome Center, The Institute of Medical Science, The University of Tokyo, Tokyo, Japan; 3 Health Intelligence Center, The Institute of Medical Science, The University of Tokyo, 4-6-1 Shirokanedai, Minato-ku, Tokyo, 108-8639 Japan; 4 Laboratory for Cancer Genomics, RIKEN Center for Integrative Medical Sciences, Suehirocho 1-7-22 E414, Tsurumi-ku, Yokohama, 230-0045 Japan; 5 BIOPIC and College of Life Sciences, Academy for Advanced Interdisciplinary Studies, Beijing Advanced Innovation Centre for Genomics, Peking University, Beijing, China; 6 Department of Internal Medicine, Seoul National University Hospital, Seoul, Korea; 7 Samsung SDS, Seoul, Korea; 8 National Cancer Research Center, Tokyo, Japan; 9 Department of Bioinformatics and Computational Biology, The University of Texas MD Anderson Cancer Center, Houston, TX USA

**Keywords:** Cancer, Cancer genomics

## Abstract

Immune reactions in the tumor microenvironment are an important hallmark of cancer, and emerging immune therapies have been proven effective against several types of cancers. To investigate cancer genome-immune interactions and the role of immunoediting or immune escape mechanisms in cancer development, we analyzed 2834 whole genome and RNA sequencing datasets across 31 distinct tumor types with respect to key immunogenomic aspects and provided comprehensive immunogenomic profiles of pan-cancers. We found that selective copy number changes in immune-related genes may contribute to immune escape. Furthermore, we developed an index of the immunoediting history of each tumor sample based on the information of mutations in exonic regions and pseudogenes and evaluated the immunoediting history of each tumor. Our immuno-genomic analyses of pan-cancers have the potential to identify a subset of tumors with immunogenicity and diverse backgrounds or intrinsic pathways associated with their immune status and immunoediting history.

## Introduction

Genome instability and the escape of immune surveillance or destruction in the tumor microenvironment are important underlying hallmarks of cancer^1^. The immune system is a large source of genetic diversity in humans and tumors^2^. Human leukocyte antigen (HLA), a vast number of unique T- and B-cell receptor genes (TCR and BCR), and somatic alterations in tumor cell genomes allow for differentiation between self and non-self (tumor) antigens via neoantigen (NAG) presentation, which contributes to positive and negative immune reactions related to cancer^3–6^. A variety of immune cells are able to infiltrate tumor tissues and suppress or promote tumor growth and expansion after the initial oncogenic process^7^. These cancer immunoediting processes^8^ determine the structure of the tumor genome via the detection and elimination of tumor cells in the early phase and are also related to the phenotype and biology of the developed cancer. It is important to investigate the escape mechanisms of tumor cells from immunoediting, and methods to observe the immunoediting history in clinical human tumors are needed.

Emerging therapies that target immune checkpoints or immune-escape molecules have proven to be effective against several types of advanced cancers^9–13^. However, most cancers remain resistant to these immunotherapies. Even after successful treatment, tumors often acquire resistance via alternative immune escape mechanisms or by acquiring genomic mutations in intrinsic immuno-signaling pathways, such as the interferon (IFN) gamma pathway or major histocompatibility complex (MHC) (HLA) presentation pathway, related to NAG^14,15^. Tumor aneuploidy is also correlated with immune escape and the response to immunotherapy^16^. Hence, in order to gain a comprehensive understanding of cancer immunology and its diversity, whole genome analysis is necessary. In this study, we analyzed the whole genome sequencing (WGS) data of 2834 donors and RNA-seq data from the Pan-Cancer Analysis of Whole Genomes (PCAWG) project in International Cancer Genome Consortium (ICGC)/The Cancer Genome Atlas (TCGA), focusing on key immunogenomic aspects using several computational approaches^17^. Here, we provide comprehensive immuno-genomic profiling data of pan-cancers that enable us to deeply investigate the relationship between tumors and immune responses. Our results demonstrate diverse genomic alterations in specific tumor types, variations in infiltrated immune cells, and T-cell receptor repertoire, as well as immunoediting during cancer development. To illustrate the immunoediting history for each cancer genome, we defined a novel immunoediting index (IEI) based on comparing exonic NAGs to antigens in pseudogenes and applied this metric to explore the underlying molecular pathways involved in immunoediting.

## Results

### Mutation landscape of immune-related genes

**Figure 1:**
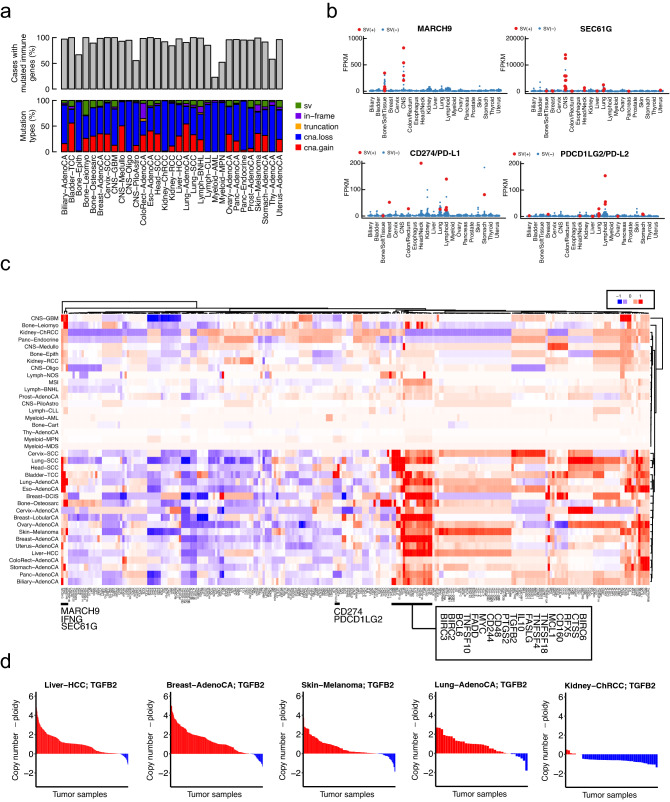
Mutation landscape of immune-related genes. (**a**) Frequency and types of somatic mutations in immune-related genes. Single nucleotide variants (SNVs), insertions and deletions (indels), structural variants (SVs), and copy number alterations (CNAs) were examined in immune-related genes and donors for multiple types of tumors, where “truncation” represents “stop gain SNV” and “frameshift indel,” and “in-frame” in frame “nonsynonymous SNV” and “in-frame indel.” (**b**) The overexpression of immune-related genes and its association with SVs in each tumor type. Red and blue dots indicate tumor samples with and without SVs, respectively. (**c**) Copy number of *IL10* offset by tumor ploidy. Tumor samples are colored red and blue to indicate whether the copy number is above or below the ploidy level, respectively. The heatmap was generated by the R software (R 3.4.0 (April, 2017)^24^). (**d**) Selective copy number changes of immune-related genes in each tumor type. Red and blue represent an excess or deficiency in the gene copy number, respectively, compared to the tumor ploidy level. The color of the element represents the mean value of the differences between copy number and ploidy.

Based on recent intensive studies of the relationship between copy number alterations (CNAs) and cancer development and progression^16,18^, CNAs have been found to be connected to the immunological profiles of cancers, although the causality is largely unknown. Somatic alterations in immune-related genes may contribute to cancer development and progression or immune escape in certain solid tumors and hematopoietic tumors. To investigate the effect of genomic alterations in the immune system, we compiled a list of 260 immune-related genes (Supplementary Table 1) assigned to one of four categories: the immune escape pathway, antigen presentation pathways for HLA class I and HLA class II, and the cytokine signaling and apoptotic pathways, including genes involved in the IFN gamma pathway. An analysis of PCAWG consensus variant calls by the PCAWG Network demonstrated that most tumor samples had at least one somatic alteration in these immune-related genes (Fig. 1a). Although CNAs were the most frequently detected type of somatic alterations, many point mutations and structural variants (SVs) were also detected in immune-related genes, including *HLA-A, HLA-B, HLA-C*, and *B2M*. Mutations in *HLA* genes are usually difficult to call because of their highly polymorphic features. Therefore, we accurately determined 367 *HLA* genotypes (class I and II, shown in Supplementary Fig. 1) using WGS data from PCAWG 2,834 donors and our new pipeline ALPHLARD (see “Methods”) and further focused on somatic point mutations in HLA genes. We identified 102 HLA somatic point mutations (75 in class I genes, 27 in class II genes) by comparing the ALPHLARD results obtained from tumor and matched-normal WGS data (Supplementary Fig. 2a). We observed that nonsense mutations and frameshift insertions and deletions (indels were concentrated near the start of class I genes, leading to loss of function. We further observed relationships between HLA somatic mutations and tumor types; for example, lymphoma (Lymph-BNHL contained a number of somatic mutations in class I genes (Supplementary Fig. 2a), while melanoma tended to acquire somatic mutations in class II genes. Colon cancer (ColoRect-AdenoCA) was characterized by recurrent indels at the start of exon 4 in *HLA-A*, a cytosine homopolymer known to be an indel hotspot^19^. This mutation was enriched in microsatellite instability (MSI)-positive tumors^20^, which was confirmed by Sanger sequencing (Supplementary Fig. 2a). In beta-2 microglobulin (B2M), which also plays a critical role in the HLA-antigen presentation machinery, we found 52 *B2M* somatic mutations, which were enriched in exon 1, specifically in Lymph-BNH and ColoRect-AdenoCA (Supplemental Fig. 2a). Overall, 3.95% (105/2658) of tumors had somatic point mutations or copy number loss in one *HLA* gene or *B2M*. *HLA* allele-specific expression is shown in Supplemental Fig. 2b. We also evaluated the RNA-seq data from healthy samples and found that nearly all allelic imbalances (two alleles of a gene are expressed at different levels) in *HLA-A*, *-B*, and *-C* occurred only in tumor samples and were possibly related to immune escape.

We also investigated SVs^21^ in immune-related genes. Although SVs are relatively rare compared to CNAs, they may have a large impact on the expression and function of affected genes, as exemplified by a recent report on the 3′-untranslated region of *CD274/PD-L1*^22^. For each immune-related gene, we compared the mRNA expression levels between SV-positive and SV-negative cases. As a result, we detected a statistically significant association between the occurrence of SVs and expression upregulation (q-value < 0.05, Fig. 1b and Supplementary Fig. 2c) in ten immune-related genes (*CD274/PD-L1*, *PDCD1LG2/PD-L2*, *MARCH9*, *IL22*, *SEC61G*, *CCND1*, *CCT2*, *INHBC*, *AKT3*, and *SOCS7*).

*SEC61G* and *MARCH9*, both of which exhibited significant overexpression related to SVs (Fig. 1b), showed different patterns from those of *TGFB2* and *IL10*. *MARCH9* showed statistical significance in some tumor types; considering the mean value of the differences in each tumor type, selective copy number gain was detected in CNS-GBM and Bone-Leiomyo. Additionally, *SEC61G* was selectively amplified in CNS-GBM and Head-SCC. Donors with SV-related overexpression and donors with selective copy number gains were highly correlated; however, selective copy number gain could only partially explain the overexpression of these genes in donors without SVs (Supplementary Fig. 3).

### CNAs of immune-related genes

In the PCAWG samples, CNAs were the most frequently observed alterations in immune-related genes (Fig. 1a). We then compared the copy numbers of immune-related genes with the ploidy levels of tumors to differentiate between selective increases in copy numbers or changes in ploidy or averaged changes in chromosomes. First, we analyzed how copy numbers changed in immune-related genes in each tumor type, including MSI-positive tumors, which are associated with strong immunogenicity^23^ because of high numbers of NAGs. For each immune-related gene, we used one-group *t*-tests to evaluate whether the copy number differences from the ploidy level were significant (the R code is shown in “Methods”). The results are summarized as a landscape of selective copy number changes, as shown in Fig. 1c (the mean copy number changes against the ploidy value) and Supplementary Fig. 4 (the statistical significance of selective copy number changes).

Next, we focused on one distinctive gene cluster that contained transforming growth factor beta 2 (*TGFB2*) and interleukin-10 (*IL10*) (Fig. 1c), which appeared to be driven by the recurrence of selective copy number gain in multiple tumor types. We examined the differences between the copy number of *TGFB2* and ploidy level and in the relative copy number of *TGFB2* for each donor of multiple tumor types (Fig. 1d). In the Liver-HCC, Breast-AdenoCA, Skin-Melanoma, and Lung-AdenoCA samples, the *TGFB2* copy number was specifically increased, rather than the ploidy level, in almost all tumors. As *TGFB2* functions as a repressor of immune cells, the amplification or gain of *TGFB2* may be at least partly related to the immune escape mechanism. By contrast, in Kidney-ChRCC, no significant selective amplification was observed.

In Lymph-NOS and Myeloid-MDS, the copy numbers of almost all immune genes were consistent with the ploidy level and were not statistically significant (minimum p = 0.498 and 0.184 for Lymph-NOS and Myeloid-MDS, respectively). MSI-positive tumors showed weak selective copy number increases for the genes in the cluster, including *IL10* (p = 0.000644); however, significant results were not observed for other immune-related genes. These results suggest that there may exist different immune escape systems in these tumor types other than the selective copy number gain of these immune-related genes.

### Immunoediting history and IEI

**Figure 2:**
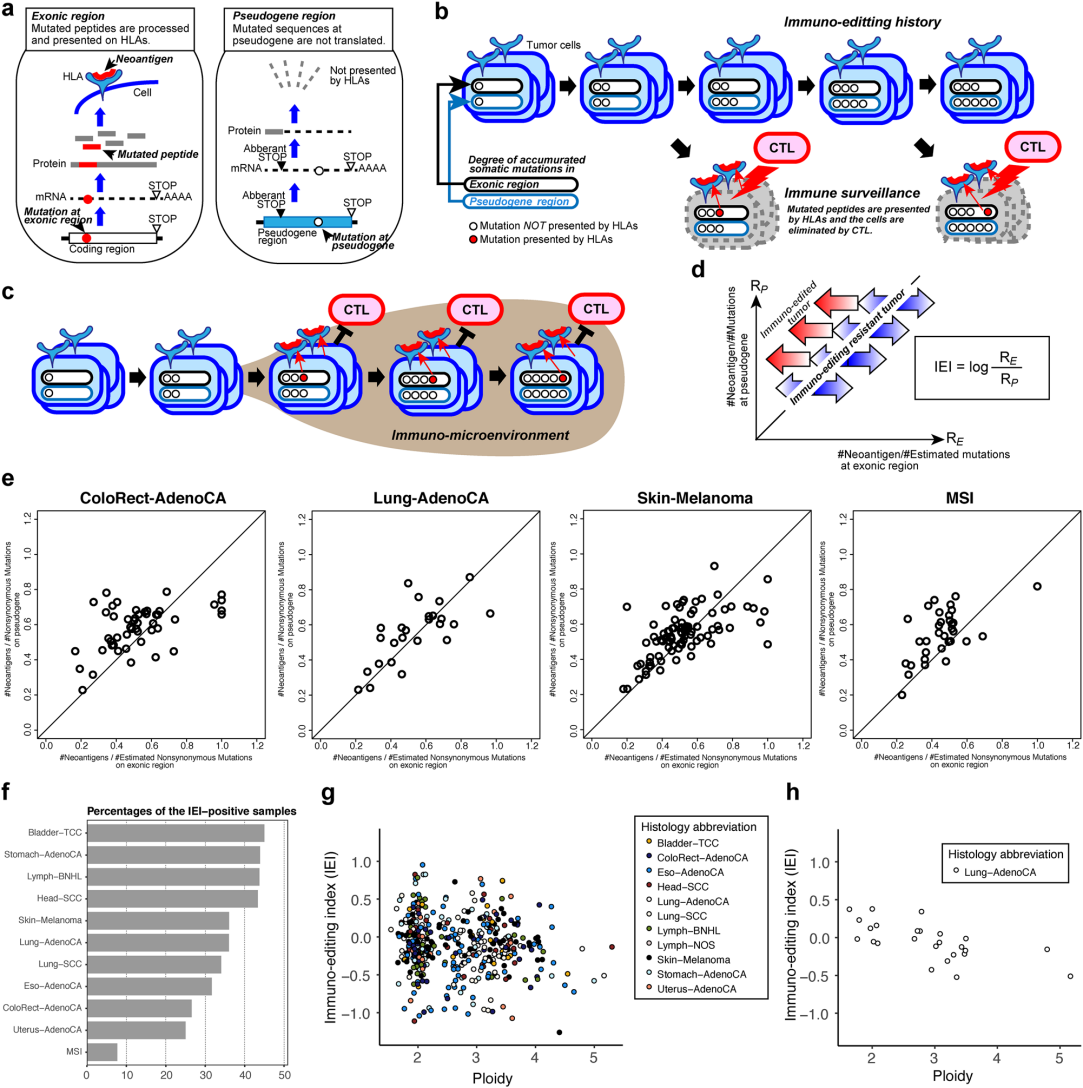
Analysis of immunoediting history. (**a**) An overview of the presentation of neoantigens (NAGs) generated from nonsynonymous mutations in exonic regions. Pseudogene regions are not translated, and mutations that accumulate in pseudogenes are not presented by the HLA complex. (**b**) Relationship between accumulated mutations in exonic regions and pseudogenes in the immunoediting history. Although cytotoxic T-cells eliminate tumor cells by recognizing these NAGs, some tumor cells escape this immune surveillance mechanism and eventually contribute to the formation of a clinical tumor. (**c**) In immunoediting-resistant tumors, the tumor cells immediately escaped from immune pressure in the carcinogenic process, and the difference between NAGs in exonic and pseudogene regions was expected to be small. (**d**) Immunopressure plot of NAGs in exonic regions and pseudogenes. The *x*-axis represents the pseudo-antigen ratio R_*P*_ for mutations in pseudogene regions, and the *y*-axis shows the neoantigen ratio R_*E*_ for mutations in exonic regions. Immunoediting index (IEI) was defined as the log ratio of R_*P*_ to R_*E*_ and was used to characterize the immunoediting history of each donor, with immunoedited and immunoediting-resistant tumors. (**e**) Immunopressure plots of four cancer types. Microsatellite instability-positive tumors show the most immunoedited tumor characteristics; in other cancers, many tumors showed an immunoediting-resistant tendency. (**f**) The proportion of immunoediting-resistant tumors. (**g**,**h**) Tumor ploidy and IEI for a pan-cancer analysis (**g**; n = 433) and lung adenocarcinoma (**h**; n = 25). Each dot represents a tumor sample.

During tumorigenesis, mutant peptides derived from nonsynonymous somatic mutations are presented by HLA molecules and recognized by T cells (Fig. 2a)^25,26^. Although these NAGs serve to eliminate tumor cells, some cells escape this immune surveillance and eventually contribute to the formation of clinical tumors (Fig. 2b)^27,28^. To estimate the strength of immune surveillance or immune pressure experienced by the tumor cells in each sample, we developed a novel approach to measure the strength of immune pressure using untranslatable pseudogenes as the internal control for each tumor (Fig. 2a) (see “Methods”). First, we identified predicted NAGs from somatic substitutions in the exonic regions of whole genome sequences. As single nucleotide variants (SNVs), indels, and SVs can be sources of NAGs, we tested all mutated peptides from these types of mutations. However, to simplify the discussion and increase the accuracy of our findings, we focused on NAGs from SNVs for subsequent analyses (Supplementary Fig. 5a and b) and also showed the NAGs from the indels (Supplementary Fig. 5c). In this process, we used the HLA types (class I and II, shown in Supplementary Fig. 1a-h) determined using our new pipeline ALPHLARD^29^. We compared them to those similarly derived from pseudogenes. The accumulation of somatic mutations in exonic regions versus somatic mutations in pseudogenes during tumorigenesis is schematically represented in Fig. 2b. For tumor cells growing under strong immune pressure, the difference between the predicted NAGs in the exonic and pseudogene regions would be large. This difference is expected to be small if tumor cells immediately escape immune pressure during the carcinogenic process (Fig. 2c). We defined the IEI according to this concept (see “Methods”). The pseudo-antigen ratio R_*P*_ for mutations in pseudogene regions and the neoantigen ratio R_*E*_ for mutations in exonic regions were plotted (Fig. 2d) to determine the immune pressure for each tumor sample. IEI was defined as the log ratio of R_*P*_ to R_*E*_, and was used to characterize the histories of different tumors, including immunoedited and immunoediting-resistant tumors.

In subsequent analyses, we investigated the history of immune pressure for multiple tumor types, as revealed by IEI. The distribution of immune pressure for the four cancers is shown in Fig. 2e. The percentage of IEI-positive samples, that is, immunoediting-resistant tumors, in each tumor type is shown in Fig. 2f. MSI-positive tumors showed immunoedited tumor characteristics, suggesting that MSI-positive tumors were under strong negative selection from the immune system. The Bladder-TCC, Stomach-AdenoCA, Lymph-BNHL, and Head-SCC samples showed immunoediting-resistant tendencies, indicating that mutations generating NAGs were removed by negative selection during tumorigenesis. We compared the IEI values with the ploidies using pan-cancer data and observed a significant negative correlation (Pearson’s correlation coefficient, *r* =  − 0.13, p = 0.0051) (Fig. 2g). Among the 11 tumor types, the strongest correlation was observed in Lung-AdenoCA (*r* =  − 0.66, p = 0.00028) (Fig. 2h), while multiple other tumor types, including ColoRect-AdenoCA, Eso-AdenoCA, and Skin-Melanoma, showed weak negative correlations, which were not statistically significant.

### Analysis of infiltrated immune cells and TCR repertoire

The study of infiltrated immune cells is important to improve our understanding of the mechanisms underlying immune escape. Based on the predicted composition of infiltrated immune cells and the expression of CD45, a pan-lymphocyte marker, we evaluated the activity of infiltrated immune cells. An example using lung cancer is shown in Supplementary Fig. 6. We focused on the activities of M2 macrophages (*y*-axis) as immune-suppressive cells and CD8^+^ T-cells (*x*-axis) as immune-effector cells, and obtained a flow cytometry-like plot for each tumor type (Supplementary Fig. 7a). In the plot, we divided the samples into four areas using the average values (shown by the dashed red lines) of M2 macrophages and CD8^+^ T-cell activities and named the areas R1 (CD8^−^/M2^+^), R2 (CD8^+^/M2^+^), R3 (CD8^+^/M2^−^), and R4 (CD8^−^/M2^−^) based on the presence or absence of M2 macrophages and CD8^+^ T cells (Supplementary Fig. 7b). The proportion of donors in each of the four areas is shown in Supplementary Fig. 7c. For over half of the donors with bone and soft tissue cancers, neither CD8^+^ T-cells nor M2 macrophages showed infiltration into the tumors. By contrast, approximately 40% of donors with stomach cancer displayed both CD8^+^ T-cell and M2 macrophage infiltration. Among the six types of tumors (lung, kidney, liver, breast, and ovarian cancers and melanoma), we observed some samples in each area (R1–R4). These were further analyzed to reveal the detailed background immune microenvironments of these areas and their diversity across tumors.

We investigated the differences in the microenvironment among the four subsets using gene set enrichment analysis (GSEA) (Supplementary Fig. 7d). Since the plot was divided into four subsets, we performed six comparisons. We observed that, in lung cancer, the inflammatory response was gradually enriched in “R1 and R3” and R2. The gene set “epithelial mesenchymal transition (EMT)” is an interesting example: EMT was found to be significantly enriched in R3 (p < 1.0E−4 for R2 vs. R3) in all comparisons between R3 and each of the other regions in kidney and lung cancers. However, in other cancer types, EMT was not significantly enriched in R3 in any of the comparisons related to R3. Activated natural killer (NK) cells were found to be candidates influencing the R3 microenvironment of kidney and lung cancers; the activities of these cells in these cancers were generally higher in R3 than in other regions (Supplementary Fig. 8).

**Figure 3:**
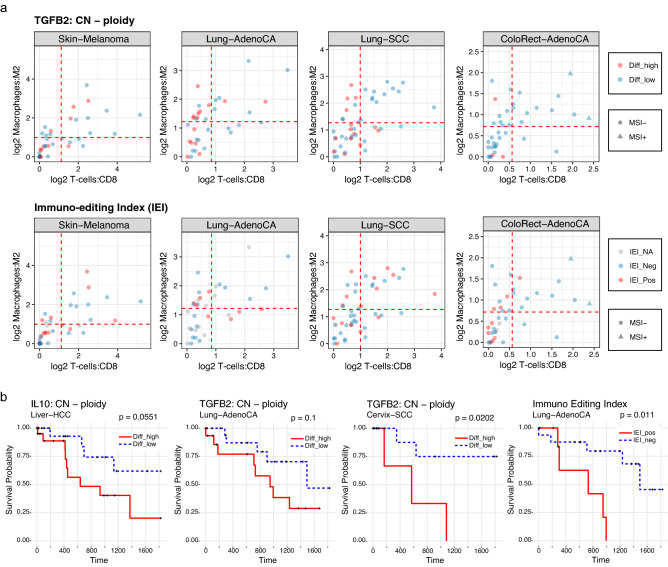
Associations among genomic alterations, immunoediting history, activities of infiltrated immune cells, and clinical outcome. (**a**) Flow cytometry-like plots representing the estimated activity of infiltrated CD8 + T-cells (*x*-axis) and M2 macrophages (*y*-axis). The dotted red line and circle represent the mean value for each axis and a sample, respectively. (**b**) Kaplan–Meier curves for overall survival were drawn using the information of selected copy number gains of *IL10* and *TGFB2* and the scores of IEI. If the score of selective copy number gain of a gene, which is defined by the difference between the copy number of the gene and the ploidy of the sample, was greater than or equal to one, the sample was classified into the Diff_high group. For the scores of IEI, the cut-off value was set as 0 for determining the IEI_Pos and IEI_Neg groups. P-values were obtained using log-rank tests.

In this study, we focused on eight types of tumors, Breast-AdenoCA, Cervix-SCC, ColoRect-AdenoCA, Liver-HCC, Lung-AdenoCA, Lung-SCC, Skin-Melanoma, and Uterus-AdenoCA, to identify associations of the immune cell infiltrations with the selective copy number gain and IEI (Fig. 3a). Lung-AdenoCA and Lung-SCC with selective copy number gains of *TGFB2* (red circles in the upper panels of Fig. 3a) showed high levels of activity for M2 macrophages and low levels of activity for CD8^+^ T-cells (statistical significance for repression of CD8^+^ T-cell in selective copy number gain tumors: p = 0.0291 and 0.0244 for the Lung-AdenoCA and -SCC, respectively); in terms of ColoRect-AdenoCA, the selective copy number gain of *TGFB2* was not observed in the majority of samples, and only a small fraction of CD8^+^ T-cell-infiltrated tumors with a selective copy number gain of *TGFB2* (p = 0.00453) was observed. By contrast, in ColoRect-AdenoCA, IEI-positive tumors were placed in the region with a small fraction of infiltrating CD8 + T cells (lower panels of Fig. 3a, p = 6.58E-4 for IEI-positive tumor CD8 + T-cell repression). In Uterus-AdenoCA, IEI-positive tumors also showed a small fraction of CD8 + T cells (p = 0.00208).

Next, we computed the RNA expression of V genes in T cell receptor (TCR) alpha; the distributions for each tumor are shown in Supplementary Fig. 9a. Although tumors had diverse median expression values, TCR expression was widely distributed within a single tumor type. The TCR repertoire was also analyzed as a key immunogenomic profile. The diversity of the TCR repertoire (alpha chain) was computed using the variety of V genes with the inverse Simpson index (Supplementary Fig. 9b). A clear correlation was observed between TCR expression and the diversity of TCR repertoire, as shown in Supplementary Fig. 9c. Notably, TCR expression and diversity were well correlated in several cancer types, including lung and skin cancers and melanoma (Supplementary Fig. 9d); however, this correlation was not observed in kidney cancer. To understand tumor characteristics related to TCR diversity, it may be necessary to focus on donors with relatively high levels of TCR expression to avoid underestimating TCR diversity as a result of insufficient number of sequence reads. *CD8A* is considered a marker of T cells, and the expression of *CD8A* is well correlated with the diversity of the TCR repertoire across tumor types (Supplementary Fig. 9e).

### Survival analysis with selective copy number gain and immunoediting history

Lastly, we analyzed the relationship between selective copy number gain (*IL10* and *TGFB2*) and overall survival, focusing on three examples: Liver-HCC, Lung-AdenoCA, and Cervix-SCC. For these tumor types, donors with selective copy number gains of *IL10* or *TGFB2* were found to have a worse overall survival than that of donors without these copy number gains (p = 0.0551 for *IL10* in Liver-HCC; p = 0.1 for *TGFB2* in Lung-AdenoCA; p = 0.0202 for both *IL10* and *TGFB2* in Cervix-SCC; Fig. 3b). We further performed a survival analysis of donors partitioned by IEI values for the aforementioned eight types of tumors and found that Lung-AdenoCA cancer donors with IEI-positive tumors (immunoediting-resistant tumors) exhibited a much worse overall survival than that of donors with IEI-negative tumors. In Lung-AdenoCA, IEI showed a possible separation (p = 0.011, Fig. 3b), whereas those for the other aforementioned gene set signatures were not significant.

## Discussion

In this study, the whole genome sequencing data and RNA sequencing data of 2,834 donors were analyzed across 31 distinct tumor types in the PCAWG project from an immunogenomic perspective, providing comprehensive immunogenomic profiles for pan-cancers, including HLA types (class I and II), NAGs from SNVs, indels, and SVs, selective copy number changes in immune-related genes, differences of infiltrated immune cells across tumor types, TCR repertoire, and immunoediting history, analyzed using a novel statistical index, IEI.

Within the mutation landscape of immune-related genes, we first focused on SVs. Although SVs in the 3-untranslated region of *CD274*/*PD-L1* are known to lead to immune escape^22^, we identified 10 genes (*CD274/PD-L1*, *PDCD1LG2/PD-L2*, *MARCH9*, *IL22*, *SEC61G*, *CCND1*, *CCT2*, *INHBC*, *AKT3*, and *SOCS7*) that showed statistically significant associations between the occurrence of SVs and the upregulation of expression. PDCD1LG2/PD-L2 can interact with PD-1 and PD-L1, resulting in inhibitory signals that modulate the magnitude of T-cell responses^30,31^. MARCH9, an E3 ubiquitin ligase, downregulates MHC class II molecules in the plasma membrane^32^, and SEC61G regulates the translocation of HLA class I proteins to the endoplasmic reticulum for clearance^33^. These findings indicate that SVs could affect HLA complexes and their expression or activity/clearance as well as immune checkpoint molecules, which may facilitate immune escape by tumor cells.

As reported in previous studies, CNAs are the most frequently observed alterations in immune-related genes. Cancers harboring many CNAs tend to show less immune involvement and poor responses to immunotherapies^16^. Hence, CNAs or genomic instability may represent a mechanism by which cytotoxic T-cells and IFN-gamma immunoedit tumors in mouse models^34^. These issues can be potentially explained by CNAs in immune-related genes. From the analysis of selective copy number changes, we found a gene cluster whose genes showed selective copy number gain in multiple tumor types, as well as chromosome *6p* loss related to HLAs. As previously reported, *TGFB2* and *IL10* were included in this cluster. *TGFB2* and *IL10* are located on chromosome *1q* and both function as suppressors of immune cells^35–37^. *IL10* is expressed not only in immune cells but also in tumors; the functions of *IL10* produced from tumor cells were mainly reported in melanoma. The selective copy number gains for these immune-related genes were likely related to tumor-immune system interactions (Fig. 1c). Recently, a molecule that simultaneously inhibited *TGFB2* and *PD-L1* expression was reported and showed high efficacy in cancer treatment^38,39^.

In Skin-Melanoma, the copy numbers of genes on chromosome 6, including HLAs, were significantly greater than the ploidy level (p = 2.26E−10 for *HLA-A*), which could paradoxically increase immune pressure. However, the copy number of *IL10* was also significantly (p = 8.1E−10) and selectively increased, potentially contributing to escape from immune pressure. By contrast, in the Kidney-ChRCC and Panc-Endocrine samples, the copy numbers of HLAs compared with the ploidy level showed the opposite tendency, and *IL10* followed this trend. As levels of HLAs are not selectively increased, the copy number gain for *IL10* may be unnecessary for immune escape. Interestingly, genomic regions containing genes that function as suppressors of the immune system, such as *TGFB2* and *IL10*, were selectively increased in many types of tumors. Copy number gains of these immune-related genes could arise and be selected during the establishment of immune escape. Therefore, selective copy number gains may be a mechanism in the history of immune escape. Based on their function, *TGFB2* and *IL10* may play important roles in immune escape. Thus, our findings indicate that selective copy number gain is a remarkable alteration in the mechanism of immune escape. However, no selective copy number gains were observed in immune checkpoint genes, that is, *PD-L1* and *PD-L2*, which function as a part of the immune escape mechanism, further suggesting the diversity of immune escape mechanisms.

We derived an index called IEI to determine the strength of immune pressure for each tumor sample. The history of immunoediting, as estimated using pseudogenes as sites free of immune pressure, indicated the existence of associations between tumorigenesis and immune escape across various tumor types. As IEI specifically focuses on HLA-binding peptides, IEI is a different concept from transcription-coupled DNA repair, which also leads to decreased mutation rates in transcribed regions in cancer^40^. As a characteristic of IEI, we observed a negative correlation between IEI and ploidy. This is most likely attributed to a scenario in which a copy number gain leads to high levels of NAG expression and, thus, high immune pressure. Recently, Eynden et al.^41^ discussed NAG depletion in various tumors. A key difference between their work and ours is that we focused on the strengthening of immune pressure for individual tumors, whereas they analyzed the characteristics of each tumor type. They concluded that the signal of negative selection is not strong or absent in most tumor types,however, interestingly, they found that only lung adenocarcinoma showed significant negative selection, which is consistent with our results.

We further investigated infiltrated immune cells to elucidate the mechanisms underlying immune escape. The characterization of sample subsets using infiltrated immune cells (Supplementary Figs. 7a and 7c) showed that different enrichment patterns of immune-related gene sets existed across tumors. EMT was enriched in R1 and R2 in melanoma and liver and ovarian cancers, whereas in kidney and lung cancers, EMT was enriched mostly in R3, that is, in the comparison of “R2 versus R3” as shown in Supplementary Fig. 7d. As the former results were consistent with our hypothesis, we further investigated the differences between R2 and R3 in kidney and lung cancers (Supplementary Fig. 7d). As shown in Supplementary Fig. 8, activated NK cells were found to be candidates influencing the R3 microenvironment. It has been previously reported that NKs are related to EMT^42^, and activated NK cells may contribute to this difference. However, further experiments will be needed to validate this hypothesis.

For the phenotypic characterization of selective copy number gain and immunoediting history (IEI), we used RNA sequencing data and overall survival. The comprehensive immune-genomic profiles of each tumor provide significant insights into immuno-oncology and a basis for the development of personalized immunotherapy.

## Conclusion

Our international collaboration team analyzed 2,834 whole genome and RNA-seq datasets across 31 distinct tumor types in PCAWG to identify key immunogenomic factors. As a result, comprehensive immunogenomic profiles of pan-cancers were generated, including HLA genotypes/mutations, neoantigens, copy number changes of immune-related genes, infiltrated immune cells, TCR repertoire, and IEI, as proposed in this paper.

## Methods

### Genomic alterations in immune-related genes in PCAWG datasets

Datasets of somatic point mutations, small indels, SVs, and CNAs were generated as part of the Pan-Cancer Analysis of Whole Genomes (PCAWG) project in ICGC/TCGA. The PCAWG-generated alignments, variant calls, annotations, and derived data sets are available for general research use, or for browsing and downloading, at http://dcc.icgc.org/pcawg/. We used the latest version of the result files on the ICGC Data Portal (https://dcc.icgc.org/releases/PCAWG/) or ICGC-TCGA Whole Genome Pan-Cancer Analysis WIKI (https://www.synapse.org/#!Synapse:syn2351328/wiki/62351).

SNV, Indel Portal

final_consensus_snv_indel_icgc.controlled.tgz

final_consensus_snv_indel_tcga.controlled.tgz

CNV

consensus.20170119.somatic.cna.annotated.tar.gz

consensus.20170217.purity.ploidy.txt.gz

Transcriptome

tophat _star_fpkm.v2_aliquot_gl.tsv.gz

SV

final_consensus_sv_vcfs_passonly.icgc.controlled.tgz

final_consensus_sv_vcfs_passonly.tcga.controlled.tgz

HLA and Neoantigen

HLA_genotype.v2.6digit.2016_0523.icgc.controlled.tsv.gz

HLA_genotype.v2.6digit.2016_0523.tcga.controlled.tsv.gz

ICGC_Neoantigen_Candidate_All.tar.gz

Overall survival

pcawg_donor_clinical_March2016_v1.xlsx

MSI

MS_analysis.PCAWG_release_v1.RIKEN.xlsx

### Bidirectional clustering

The bidirectional clustering was performed in R using the ComplexHeatmap library (Fig. 1c and Supplementary Fig. 4). The source code was as follows:
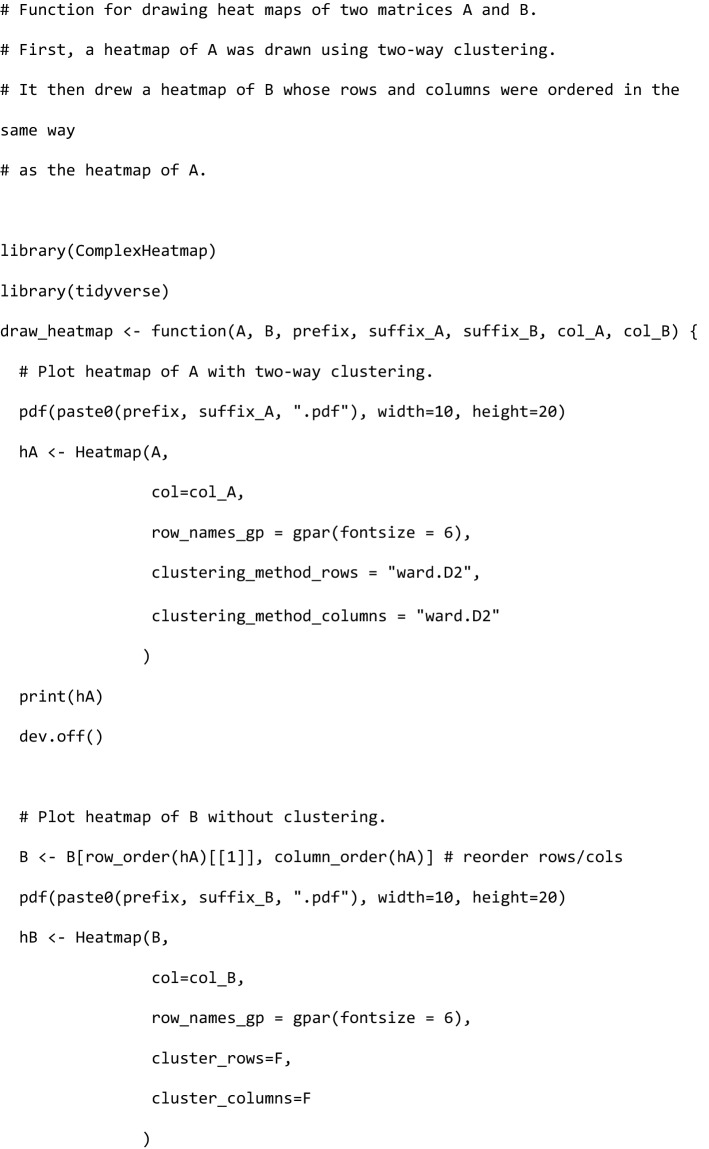

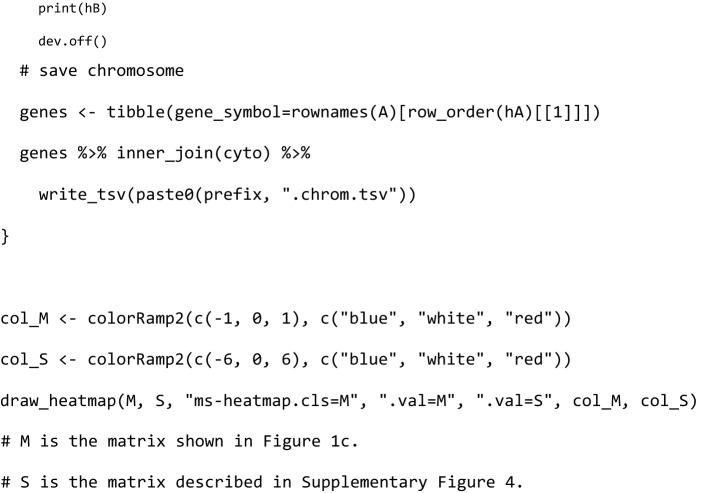


This R code was used to perform a biclustering algorithm on matrix A, and the orders of the samples and genes after clustering were used to represent matrix B.

### GSEA

In each tumor type, four regions, R1 (CD8^−^/M2^+^), R2 (CD8^+^/M2^+^), R3 (CD8^+^/M2^−^), and R4 (CD8^−^/M2^−^) (Supplementary Fig. 7b) were used to divide the samples into four groups. For gene sets of MsigDB, six GSEA analyses were performed for each tumor type: (1) “R1 vs R2,” (2) “R2 vs R3,” (3) “R3 vs R4,” (4) “R1 vs R3,” (5) “R1 vs R4”, and (6) “R2 vs R4.” In the GSEA analysis, the multiplicity of the testing was adjusted to 10,000 permutations.

### Immune cell components

For CIBERSORT implementation, FPKM values were used after upper-quartile normalization as input gene expression values (FPKMs were in linear space, without log-transformation), and the default LM22 was used as the signature gene matrix. Twenty-two leukocyte fractions were imputed using CIBERSORT. Originally, CIBERSORT was proposed for RNA expression data obtained using microarray analysis. However, CIBERSORT has also been used in bulk tumor RNA-seq^43,44^ and single-cell RNA-seq^45^. The correlation between the results obtained using microarray data and RNA-seq data from 166 LAML-US tumors was evaluated independently,the observed correlation coefficient was 0.93, which was significantly high. Therefore, CIBERSORT was applied to the RNA-seq data (Supplementary Fig. 10).

### NAG prediction

From the PCAWG preliminary consensus files, 2,786 annotated .tsv files were generated using ANNOVAR, and exclusion samples were removed according to release_may2016.v1.3.tsv. We generated neo-peptides (candidate neoantigens), which cannot appear in wild-type protein sequences, generated by single nucleotide nonsynonymous mutation (non-synonymous SNV), indel in the exonic region, and structural variation (SV). For nonsynonymous SNVs, the corresponding mutant/wild-type peptides of length 8–11-mer, including an amino acid substitution, were constructed using the UCSC RefSeq mRNA and refFlat data (http://hgdownload.soe.ucsc.edu/downloads.html). For in-frame indel mutations, where the downstream peptide sequence does not change, mutant peptides were generated in the same manner as that of nonsynonymous SNVs. For out-of-frame indel mutations, a mutant peptide sequence was generated from the mutation position to the position where the stop codon first appeared, and 8–11 mer peptides, including a part of the mutant peptide region, were generated. For SVs, we considered the following five cases that express the following candidate neoantigens, namely, combinations of: (1) 5’ exon and 3’ exon, (2) 5’ exon and 3’ intron, (3) 5’ exon and 3’ intergenic, (4) 5’ intron and 3’ exon, and (5) 5’ intron and 3’ intron. For example, the 5’ exon refers to the exon including the join region on the 5’ upstream gene of the fusion gene. In the cases of (1), (4), and (5), if they were in-framed, 8–11 mer mutant peptides around the joint position that spanned both gene regions were generated. Otherwise, as the generated sequence was out-of-frame or did not appear in wild-type, 8–11 mer mutant peptides were generated in the same manner as those generated by indel mutation. In the case of (4), we skipped incomplete 3’ exons, including the join region, and used a combination of the 5’ upstream exon and complete downstream 3’ exon.

The binding affinities (IC_50_) of all generated peptides were predicted using netMHCpan3.0^46^ for HLA class I and netMHCIIpan3.1^47^ for HLA class II. Lastly, neoantigens were counted for each patient by considering mutant peptides with IC_50_ values below 500 as neoantigens. Here, neoantigens were counted as the number of mutations that can generate neoantigens; thus, each mutation was counted once, even if more than one neoantigen was generated for one or more HLAs. It is worth noting that mutations in which the annotated information was not consistent with UCSC RefSeq mRNA and refFlat data were omitted as database mismatches. The ratio of the number of non-skipped nonsynonymous mutations to the number of all observed nonsynonymous mutations was defined as the concordance rate. Although this value was nearly 1 in all cases (greater than 0.99, on average), it was used as a tuning parameter, as described below.

### IEI

To evaluate the sample-specific immunoediting history, an immunoediting index (IEI) describing the degree of accumulated immune suppression was established. IEI compared the ratio of the number of neoantigens to the number of nonsynonymous mutations in exonic regions and in the control regions, which were not affected by immune pressure. Pseudogene regions were used as internal controls for a tumor, and only pseudogene mutations whose genomic positions were downstream of the stop codon were extracted according to PseudoPipe v.74 (http://www.pseudogene.org/pseudopipe/). The following assumptions were made: (1) nonsynonymous mutations in exonic regions can be suppressed by immune pressure if their mutant peptides can bind to HLAs, and (2) synonymous mutations in exonic regions and nonsynonymous/synonymous mutations in pseudogene regions are not affected by immune pressure. Under these assumptions, the number of nonsynonymous mutations in exonic regions could be lower than the number of ideal nonsynonymous mutations in exonic regions, indicating the hypothetical number of nonsynonymous mutations under non-immune pressure. Several quantities were defined, as follows:

・ Number of nonsynonymous mutations used to evaluate neoantigens (not skipped by database mismatch) in exonic regions = #nonsynE

・ Number of synonymous mutations in exonic regions = #synE

・ Number of predicted neoantigens in exonic regions = #NagE

・ Number of nonsynonymous mutations used to evaluate neoantigens (not skipped by database mismatch) in pseudogene regions = #nonsynP

・ Number of synonymous mutations in pseudogene regions = #synP

・ Number of predicted neoantigens in pseudogene regions = #NagP

・ Concordance rate of mutation annotations in exonic regions = ${c}_{\mathrm{exon}}$

・ Concordance rate of mutation annotations in pseudogene regions = ${c}_{\mathrm{pseudo}}$

In the calculation of IEI, we did not include mutations in the XY chromosome and NAGs generated from them. The number of nonsynonymous mutations in the exonic region was adjusted to obtain the number of ideal nonsynonymous mutations (#*InonsynE*) using the above quantities as follows:

$$\#InonsynE =\frac{{c}_{\mathrm{exon}}}{{c}_{\mathrm{pseudo}}} \times \#synE \times \frac{\#nonsynP}{\#synP},$$

where #*InonsynE* was set to #*NagE* if #*InonsynE* was less than #*NagE*.

IEI was calculated as the modified log ratio in terms of the numbers of neoantigens and nonsynonymous mutations, and was equal to the sum of the numbers of neoantigens and non-neoantigens between exonic and pseudogene regions as follows:

$$IEI = \log \frac{{(\# {\text{NagE + C})}/(\# {\text{InonsynE + C})}}}{{(\# {\text{NagP + C})}/(\# {\text{nonsynP + C})}}},$$

where *C* is a regularized constant, set to 0.5, for the analysis. To obtain robust results, we prepared the following exclusion criteria: (1) #nonsynE = 0 and/or #synE = 0, (2) the sum of #nonsynE and #synE is less than 50, and (3) #nonsynP < 5 and/or #synP < 5. The samples that met one or more of the exclusion criteria were excluded from the analysis of IEI.

### Pseudogene selection

PseudoPipe (build 74)^48^ was used as a pseudogene database for analysis, which included the region and the parental gene of each pseudogene, among other information. First, pseudogene mutations in each sample were extracted from the VCF file based on the pseudogene regions described in PseudoPipe. Next, each pseudogene in PseudoPipe was aligned to the parental gene using Clustal Omega (version 1.2.1)^49^ with default settings. Each pseudogene mutation was converted to a parental gene mutation located at the same position as that of the pseudogene mutation in the alignment. Pseudogene mutations were excluded from neo-antigen analysis if the position corresponded to an intron of the parental gene, or if the bases differed at the position in the alignment of the pseudogene and the parental gene. Thus, except for the above cases, pseudogene mutations were treated as exonic mutations. An immunoediting history analysis was applied to the converted mutations, and the results were used as an internal control. Mutations in the pseudogene regions were used directly without information on parental genes. However, the amino acid composition in pseudogene regions with parental genes was considered similar to that in the exonic regions. Additionally, in pseudogene regions, many stop codons were presented, and a method was determined to handle these. Therefore, pseudogene regions with parental genes were used as suitable internal controls to evaluate the strength of immune pressure.

### Survival analysis

Kaplan–Meier curves for overall survival were drawn using the R function, ggkm (provided in https://statbandit.wordpress.com/2011/03/08/an-enhanced-kaplan-meier-plot/); the source code of the ggkm function is shown on the above website. We defined two groups of samples based on the information of selected copy number gains of IL10 and TGFB2 and the scores of IEI. For selective copy number gain, we classified the sample into the Diff_high group if the score of selective copy number gain of a gene was greater than or equal to one. Here, the score of the selective copy number gain was defined by the difference between the copy number of the gene and the ploidy of the sample. For IEI, the cut-off value was set to 0 to determine the IEI_Pos and IEI_Neg groups. The difference between the two Kaplan–Meier curves was evaluated using log-rank tests, which were performed using the survdiff function in R.

## Supplementary Information

Supplementary Figure 1.

Supplementary Figure 2.

Supplementary Figure 3.

Supplementary Figure 4.

Supplementary Figure 5.

Supplementary Figure 6.

Supplementary Figure 7.

Supplementary Figure 8.

Supplementary Figure 9.

Supplementary Figure 10.

Supplementary Table 1.

Supplementary Information.
