# Supplementary figures and images for: Immunogenomic pan-cancer landscape reveals immune escape mechanisms and immunoediting histories
Source: Sci Rep. 2021 Aug 3;11:15713. doi: 10.1038/s41598-021-95287-x (PMC8333422; doi:10.1038/s41598-021-95287-x)

# Supplementary Figure 1

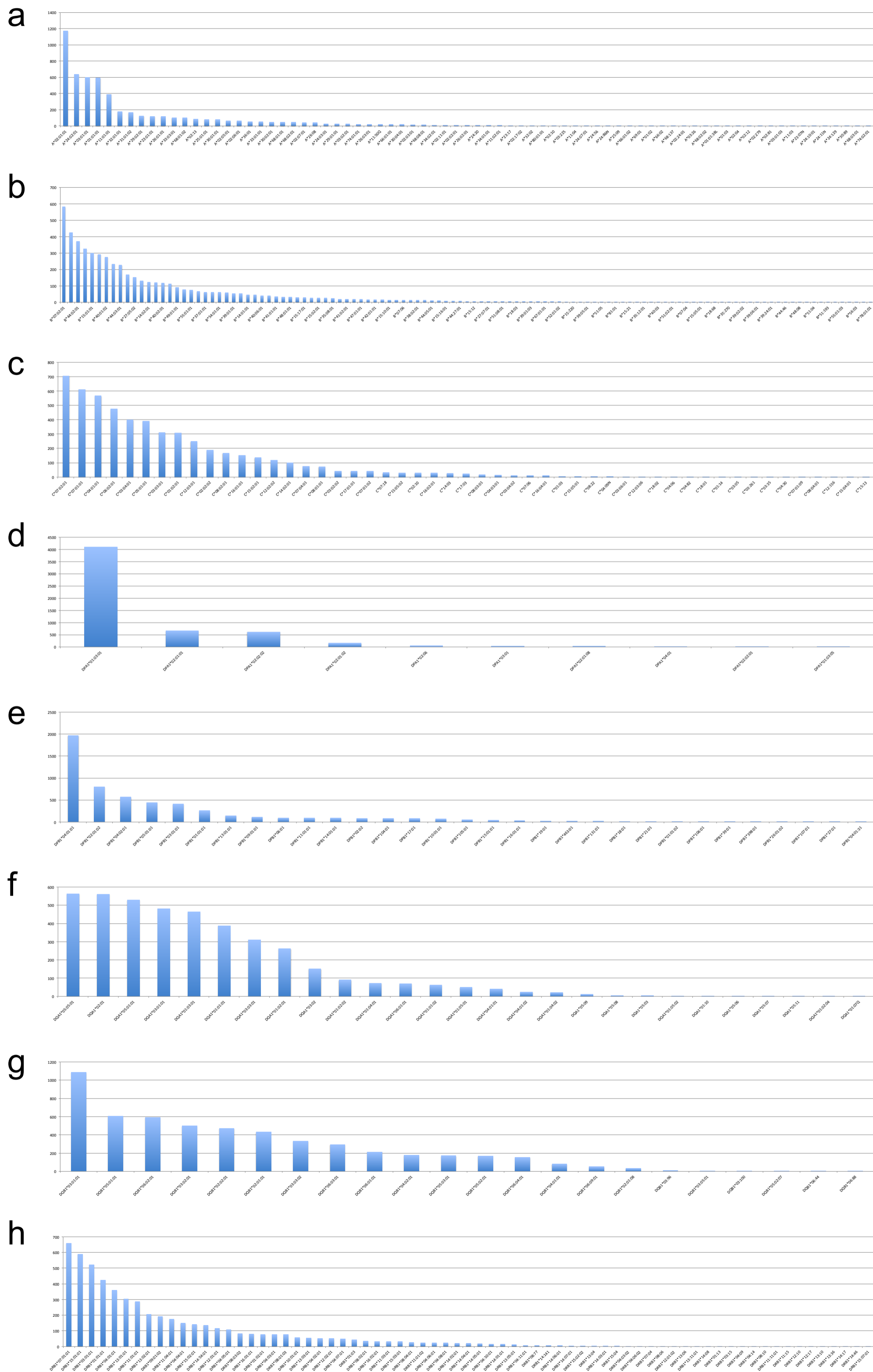

Supplement: Supplementary file 1 — Supplementary Figure 1. [file 41598_2021_95287_MOESM1_ESM.pdf]

Supplementary Figure 2

a

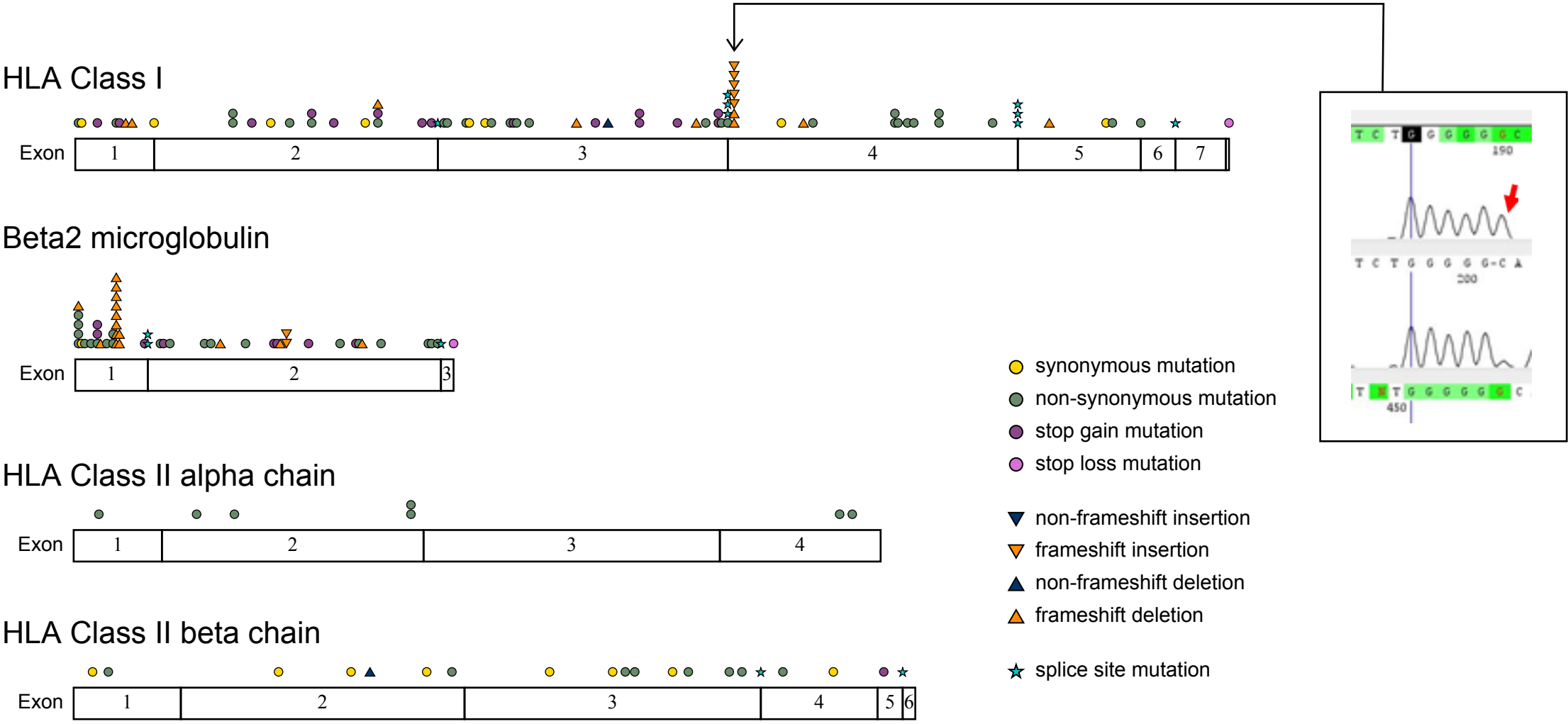

b

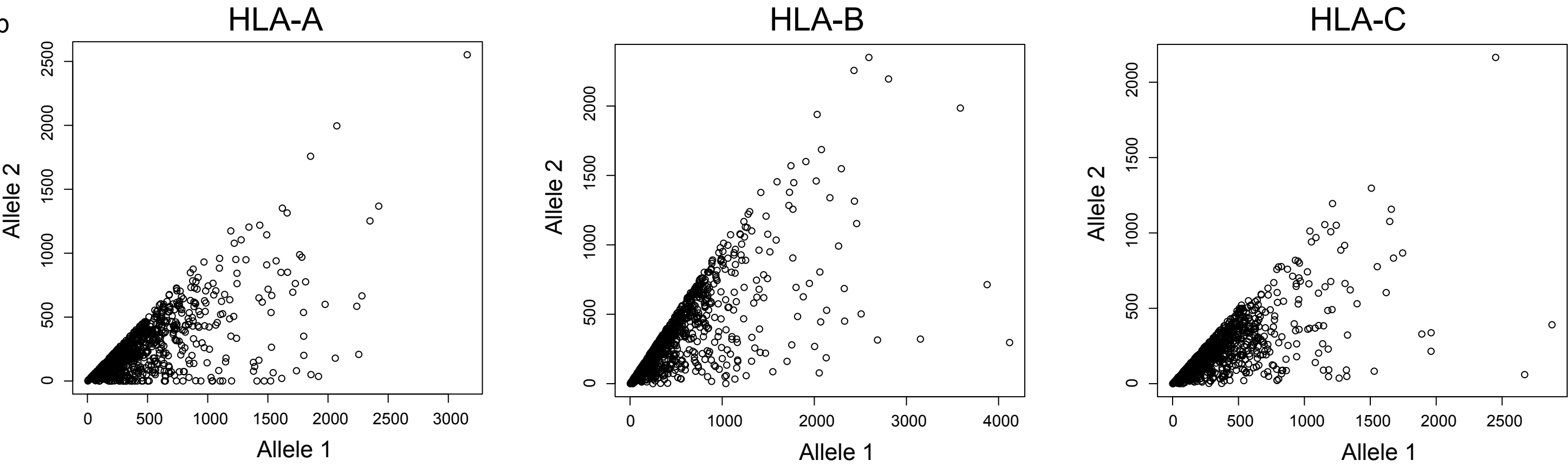

c

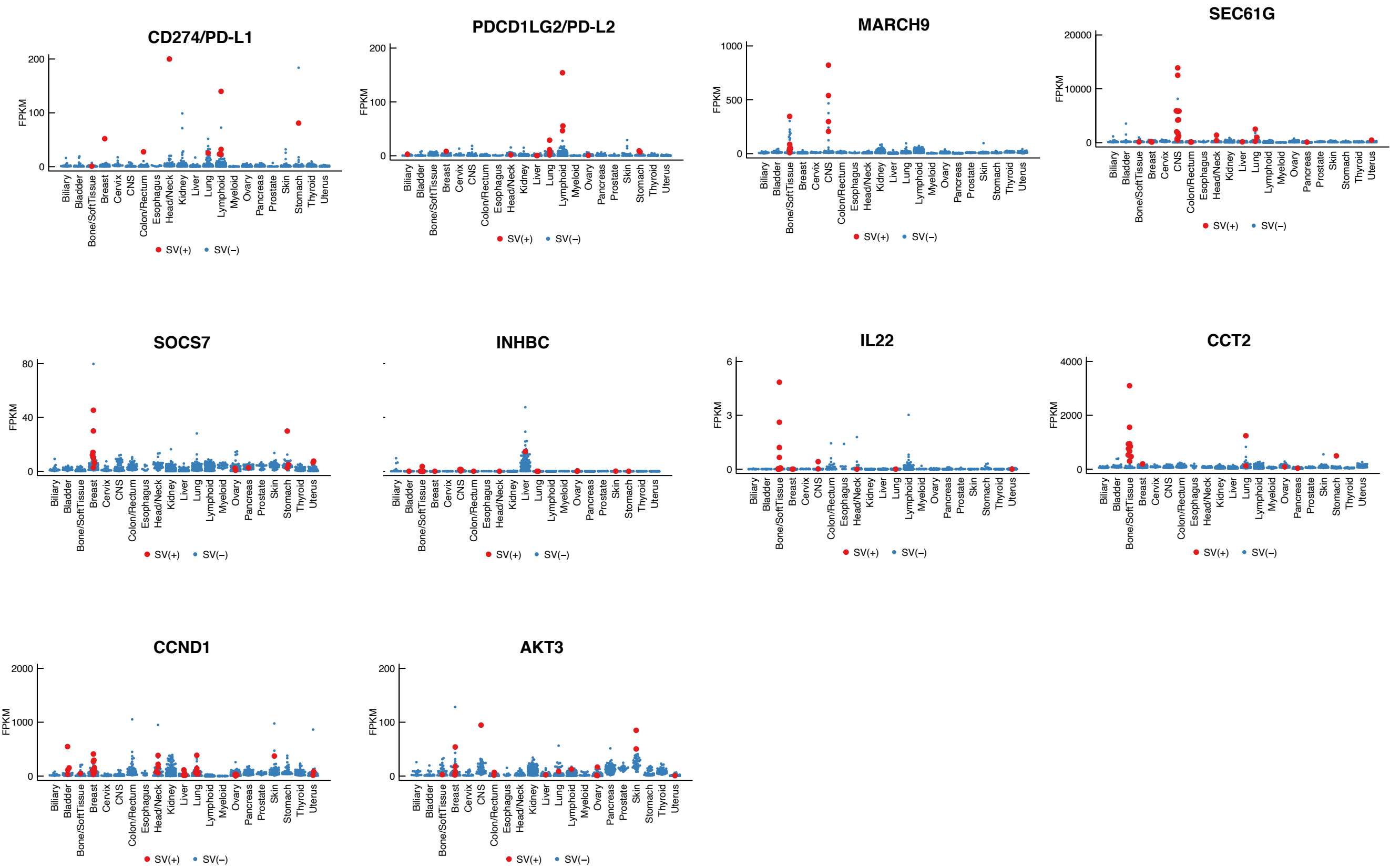

Supplement: Supplementary file 2 — Supplementary Figure 2. [file 41598_2021_95287_MOESM2_ESM.pdf]

Supplementary Figure 3

MARCH9

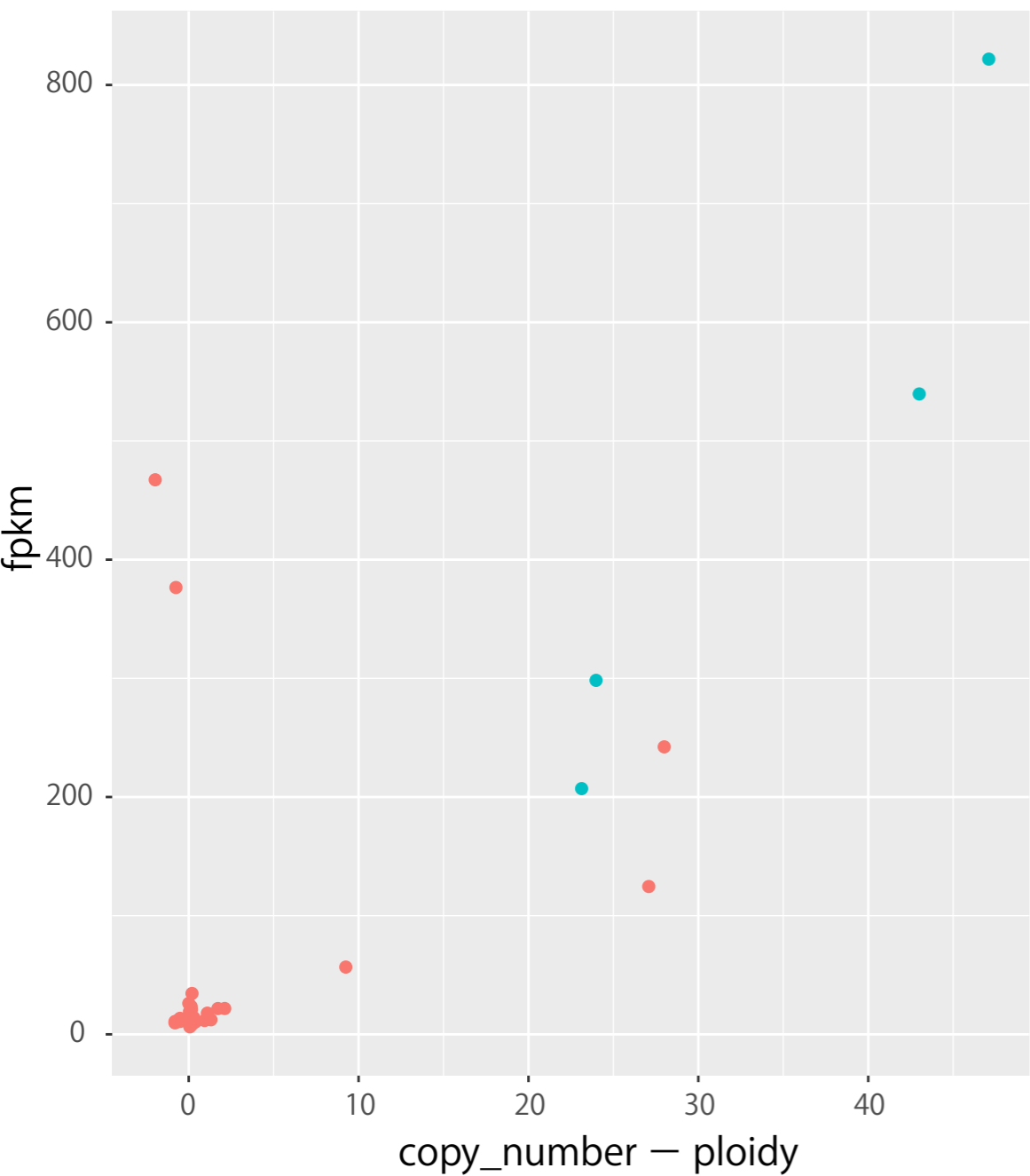

SEC61G

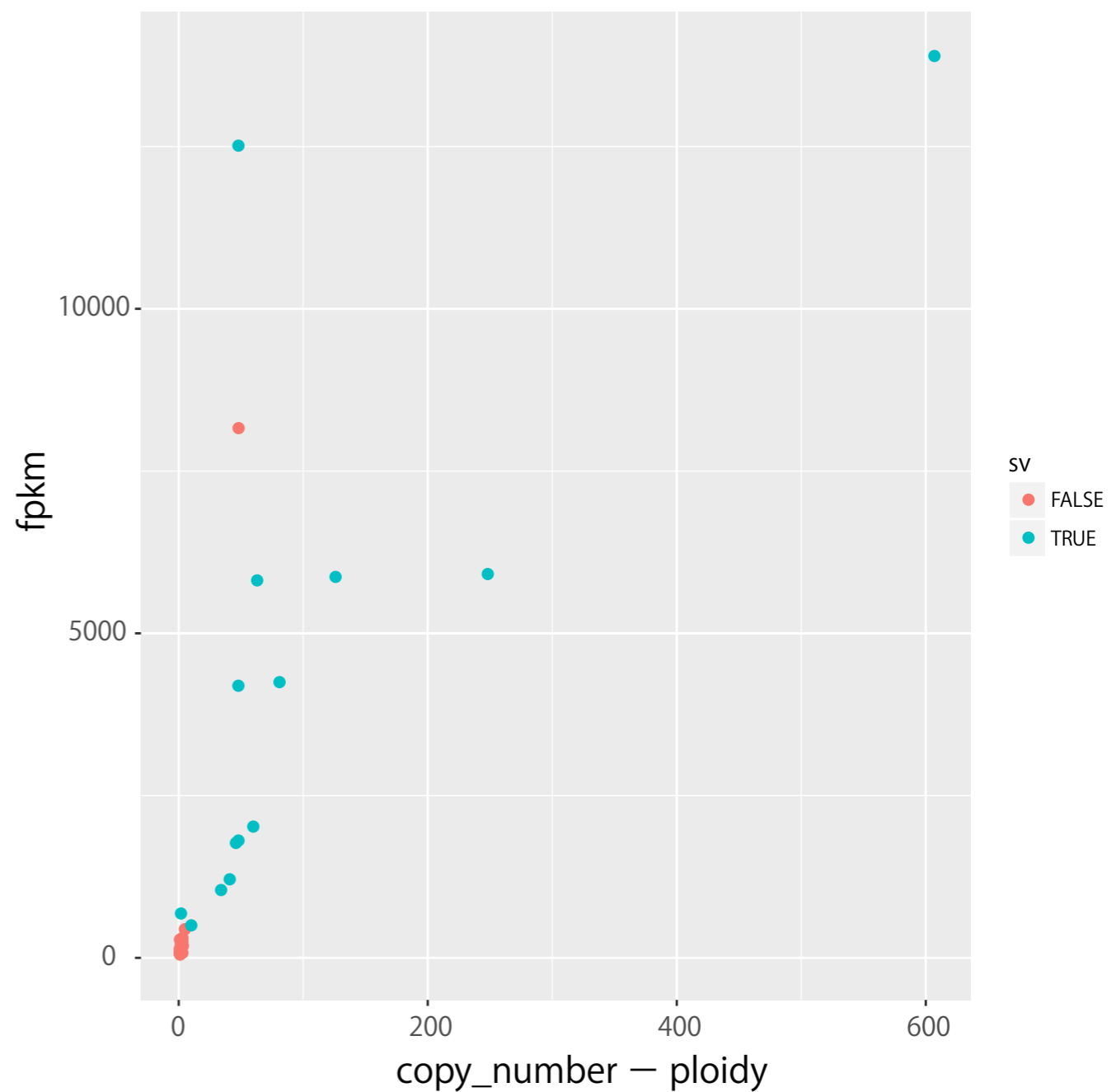

Supplement: Supplementary file 3 — Supplementary Figure 3. [file 41598_2021_95287_MOESM3_ESM.pdf]

## Supplementary Figure 4

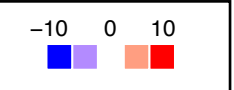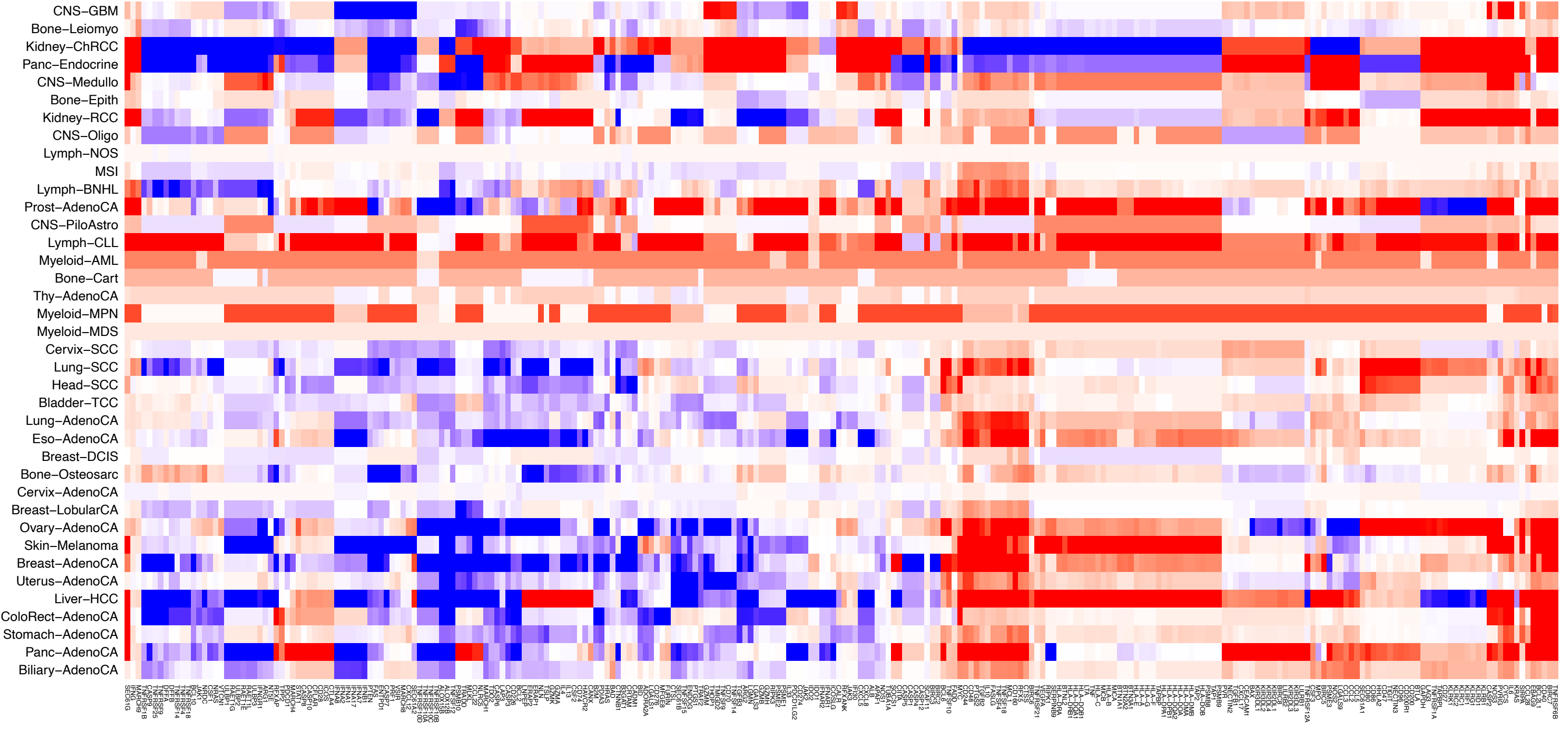

Supplement: Supplementary file 4 — Supplementary Figure 4. [file 41598_2021_95287_MOESM4_ESM.pdf]

Supplementary Figure 5

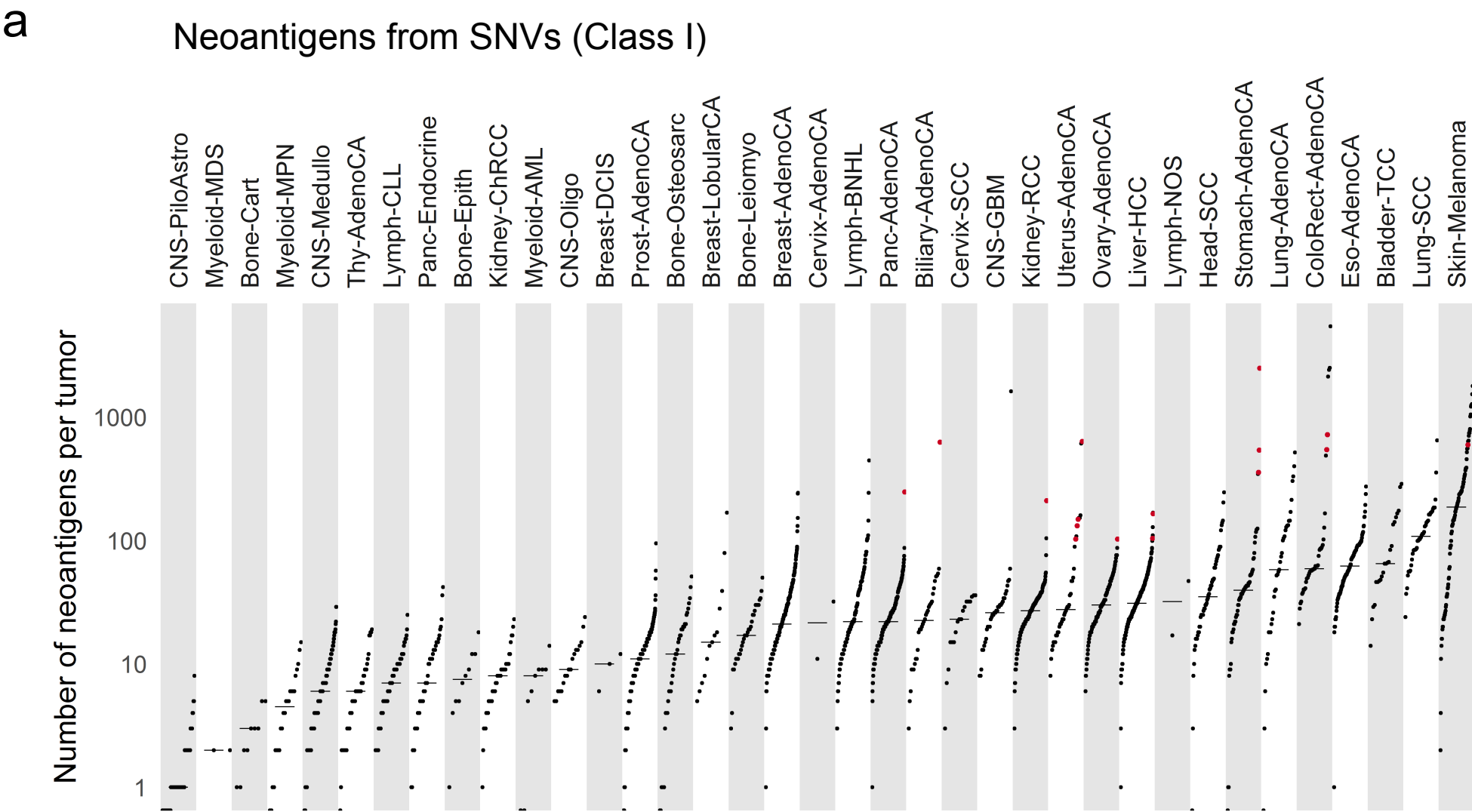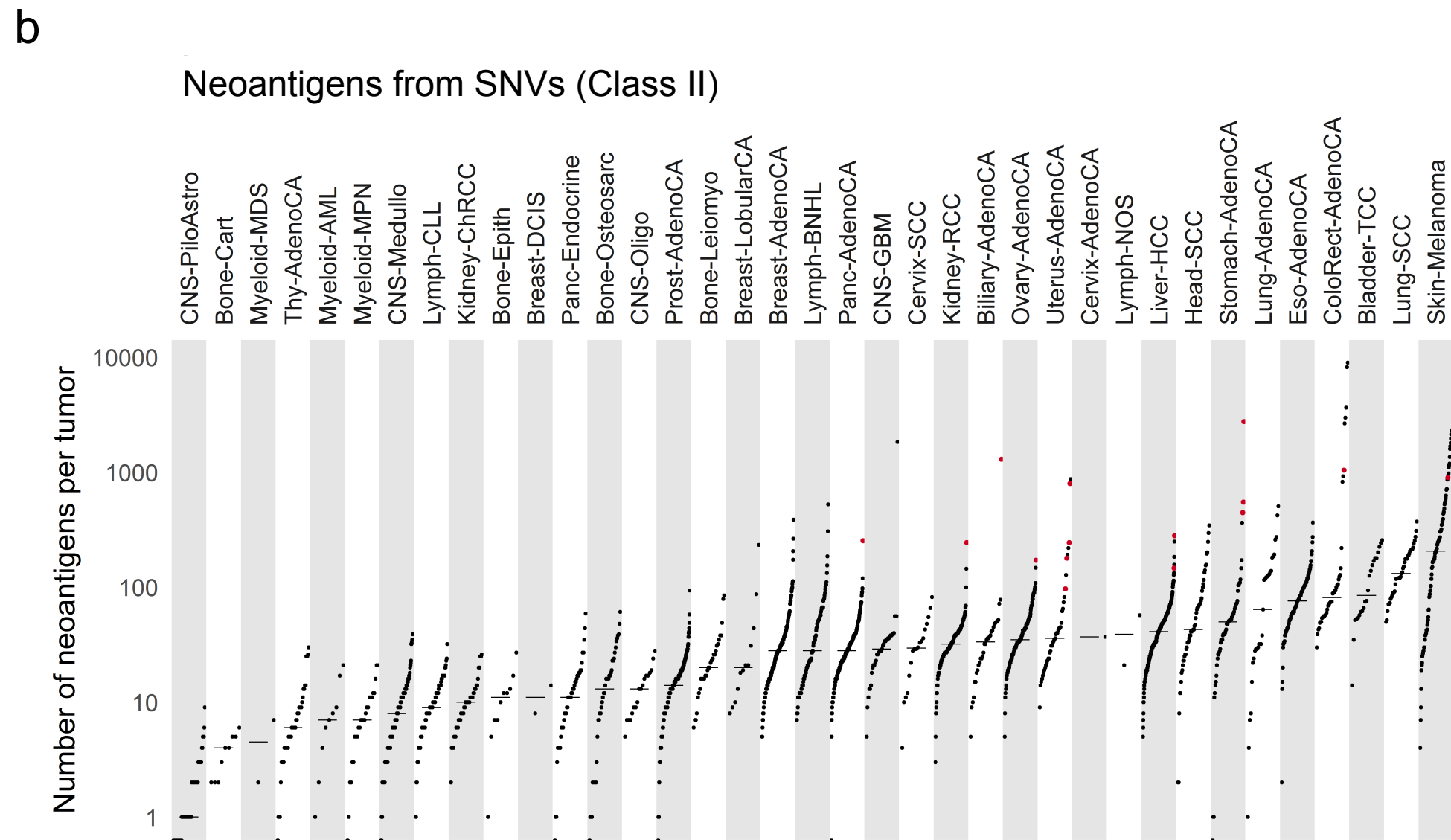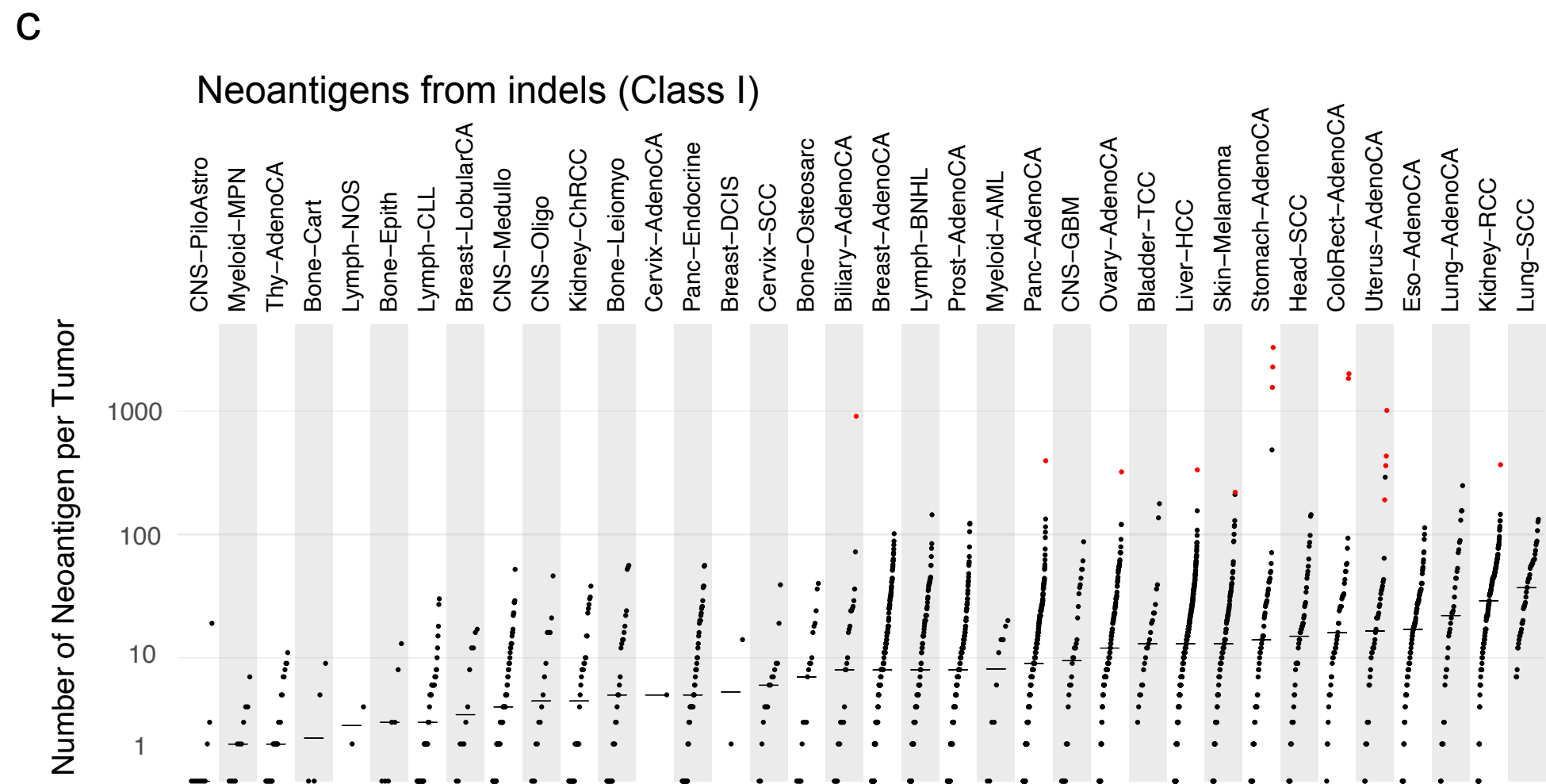

Supplement: Supplementary file 5 — Supplementary Figure 5. [file 41598_2021_95287_MOESM5_ESM.pdf]

# Supplementary Figure 6

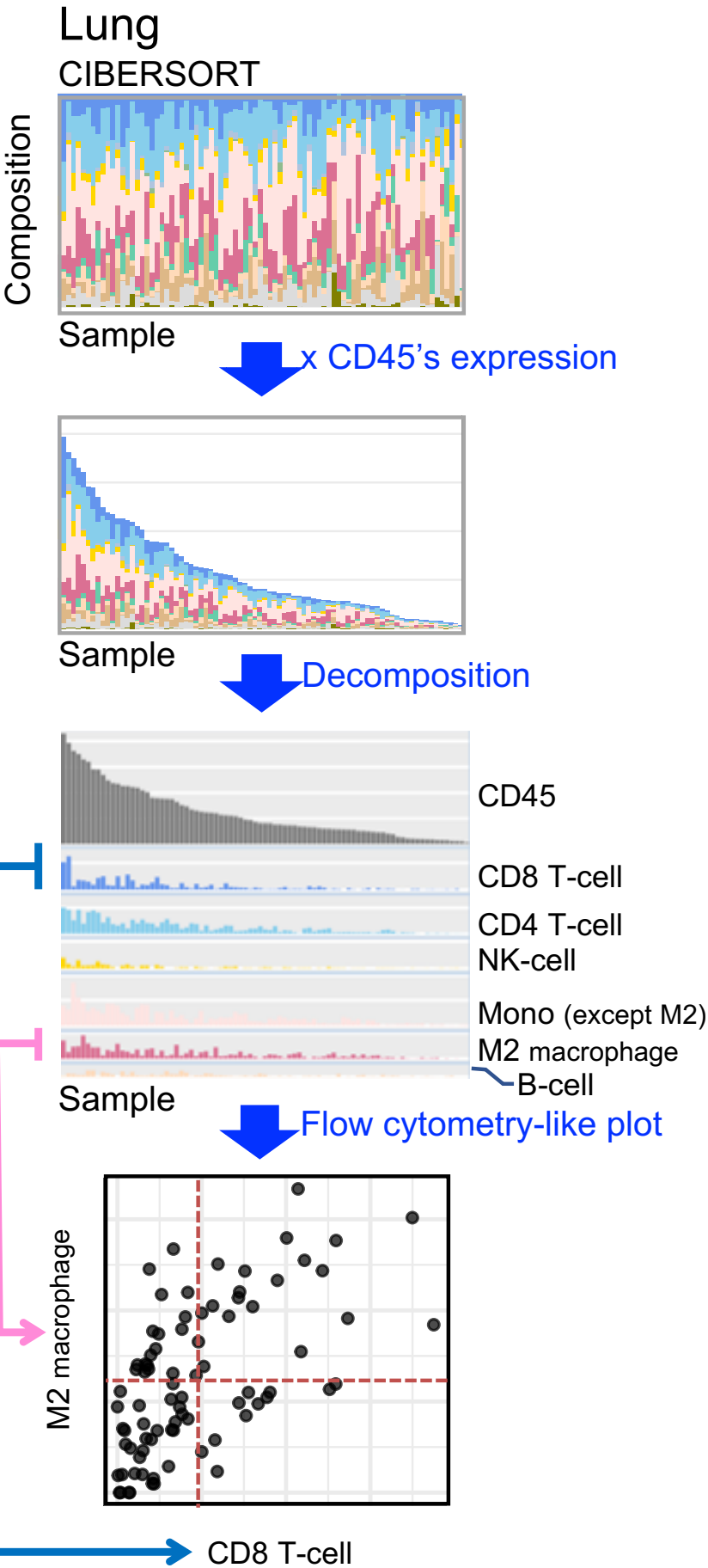

Supplement: Supplementary file 6 — Supplementary Figure 6. [file 41598_2021_95287_MOESM6_ESM.pdf]

Supplementary Figure 7

a

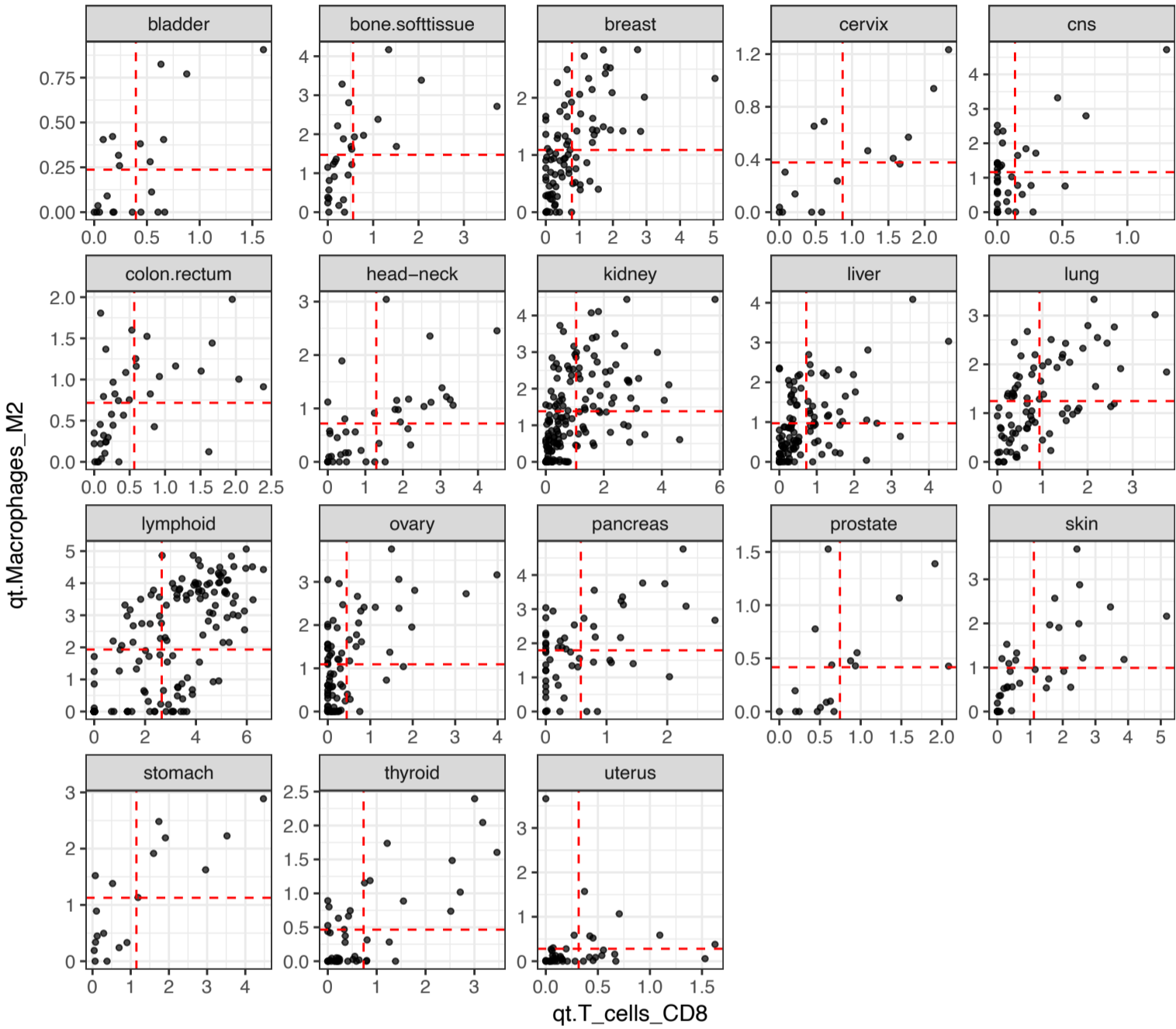

b

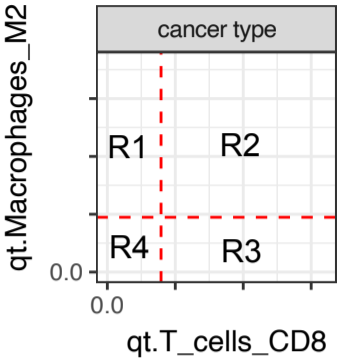

c

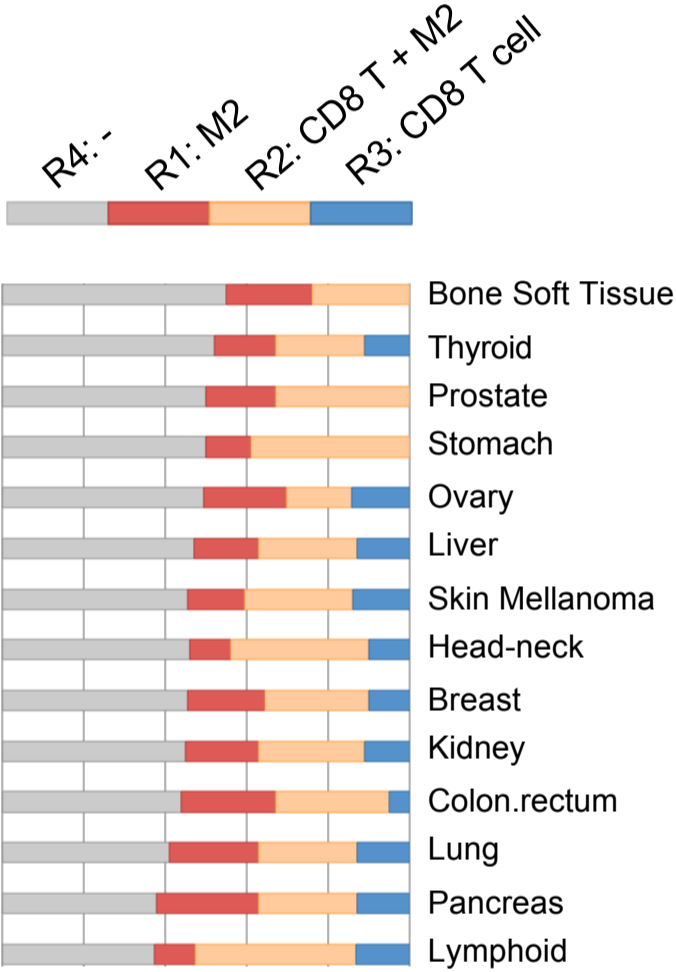

d

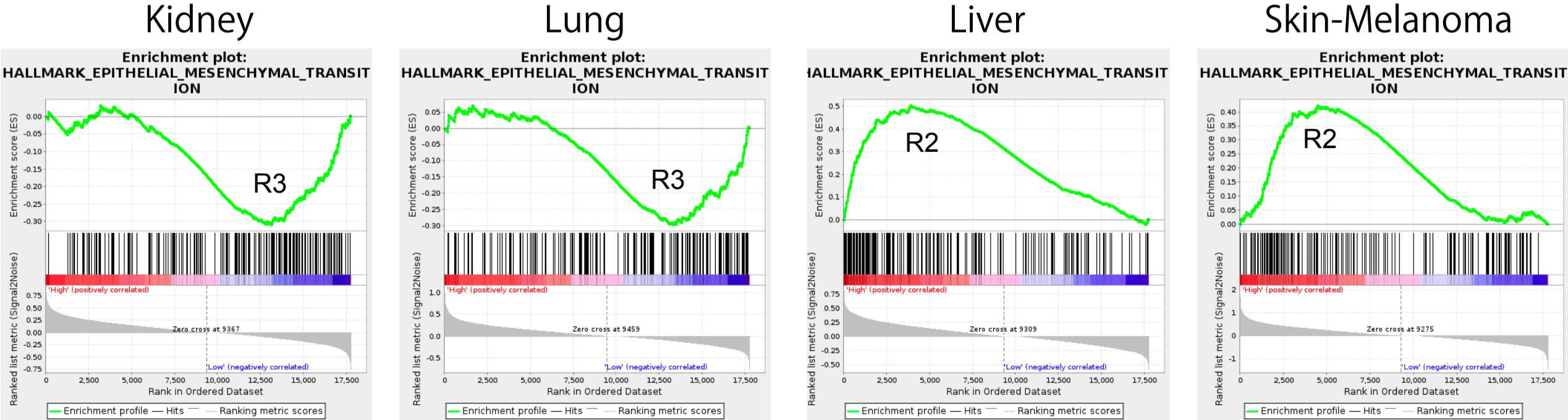

Supplement: Supplementary file 7 — Supplementary Figure 7. [file 41598_2021_95287_MOESM7_ESM.pdf]

Supplementary Figure 8

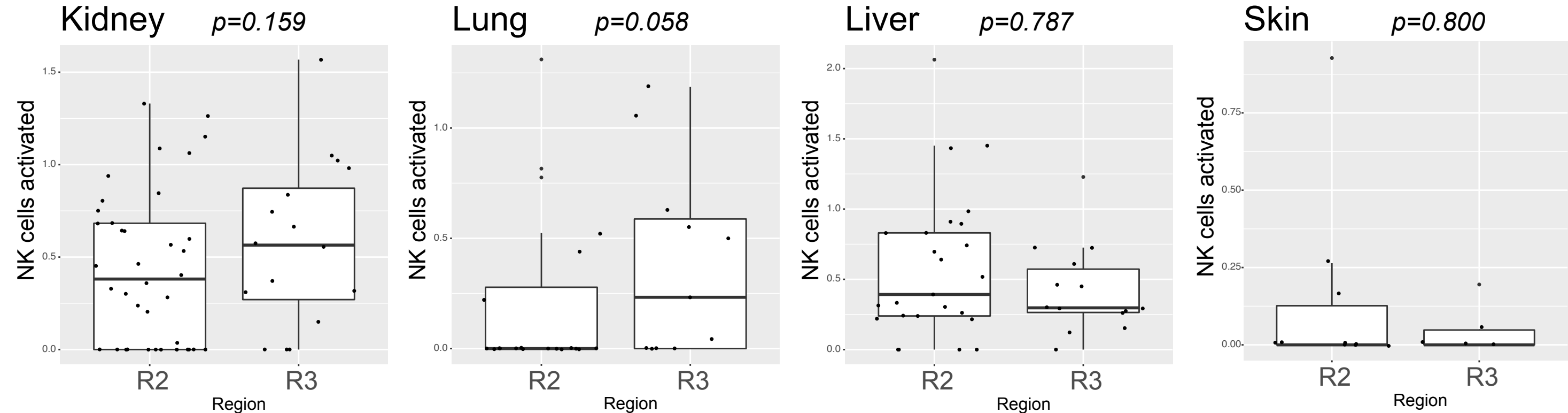

Supplement: Supplementary file 8 — Supplementary Figure 8. [file 41598_2021_95287_MOESM8_ESM.pdf]

Supplementary Figure 9

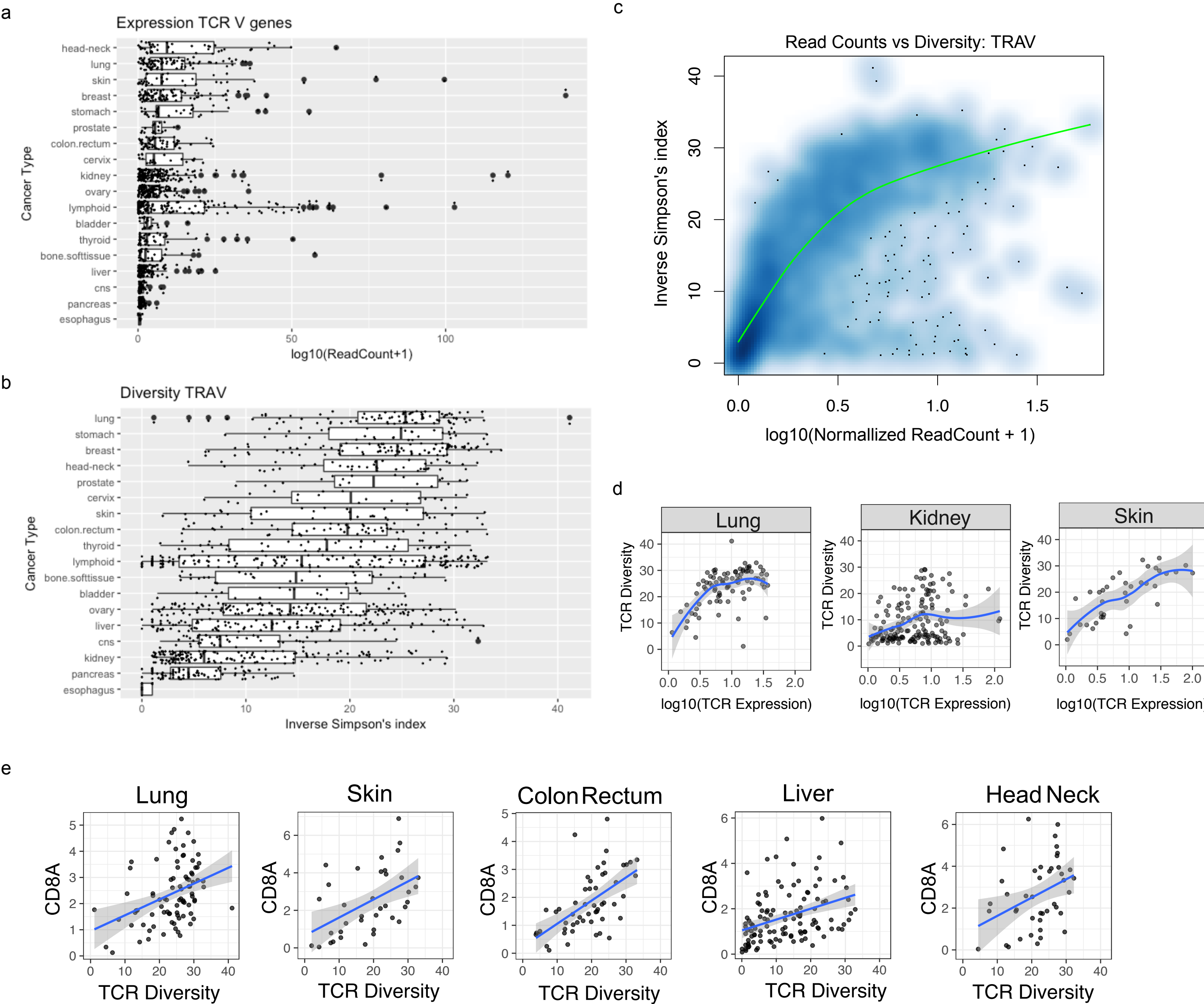

Supplement: Supplementary file 9 — Supplementary Figure 9. [file 41598_2021_95287_MOESM9_ESM.pdf]

# Supplementary Figure 10

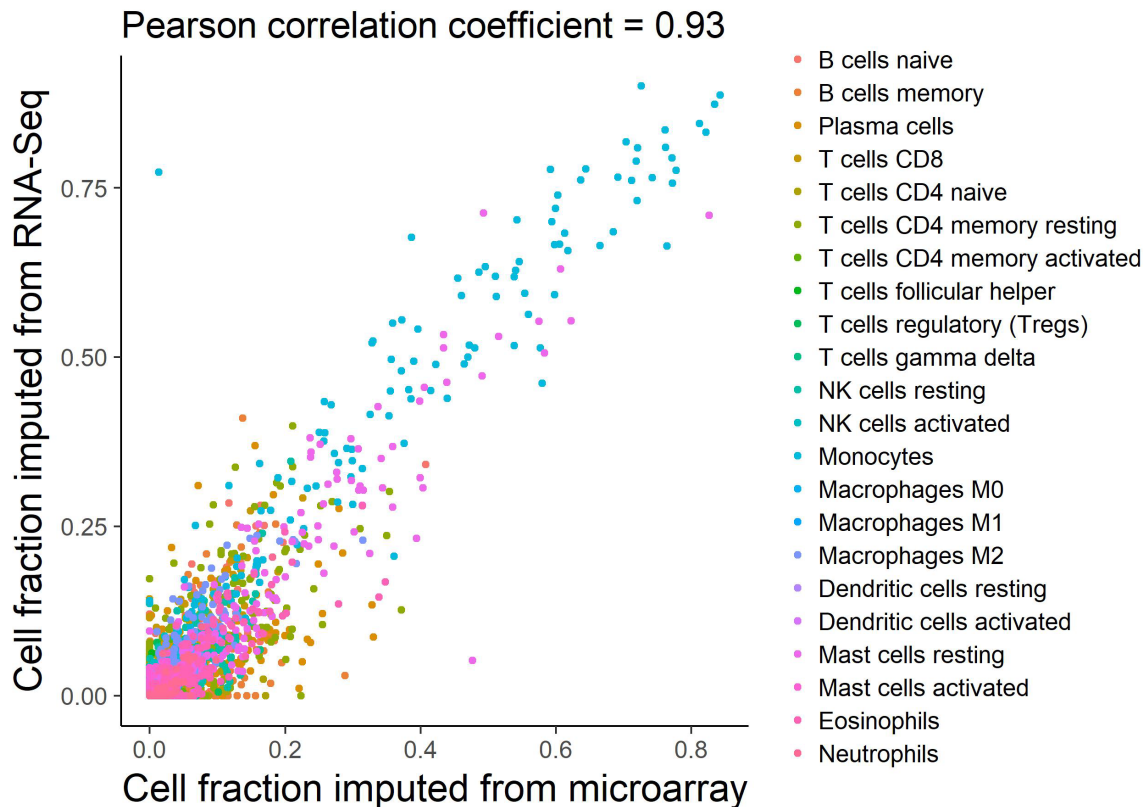

Supplement: Supplementary file 10 — Supplementary Figure 10. [file 41598_2021_95287_MOESM10_ESM.pdf]
