# Supplementary material for: Immunogenomic pan-cancer landscape reveals immune escape mechanisms and immunoediting histories
Source: Sci Rep. 2021 Aug 3;11:15713. doi: 10.1038/s41598-021-95287-x (PMC8333422; doi:10.1038/s41598-021-95287-x)
Supplement: Supplementary file 11 — Supplementary Table 1. [file 41598_2021_95287_MOESM11_ESM.pdf]

Supplementary Table 1

ADORA2A	CASP9	DFFB	IFI30	LNPEP	RAET1L	TNFSF14
ALB	CCL2	EBAG9	IFNA1	LTA	RFX5	TNFSF15
ALOX12B	CCL22	ENDOG	IFNA17	MARCH1	RFXANK	TNFSF18
ALOX15B	CCL28	ENTPD1	IFNA2	MARCH4	RFXAP	TNFSF4
APAF1	CCL3	ERAP1	IFNA7	MARCH8	RIPK1	TNFSF9
ARG1	CCL5	ERAP2	IFNA8	MARCH9	RIPK3	TPP2
ARG2	CD160	FADD	IFNAR1	MCL1	SCAF11	TRADD
B2M	CD200	FAS	IFNAR2	MFGE8	SEC61A1	TRAF2
B3GAT1	CD200R1	FASLG	IFNB1	MICA	SEC61A2	TSLP
BAD	CD226	FURIN	IFNG	MICB	SEC61B	TUBA1A
BAX	CD244	GAPDH	IFNGR1	MLKL	SEC61G	ULBP1
BCL10	CD27	GZMA	IL10	MPO	SERPINB9	ULBP2
BCL2	CD274	GZMB	IL13	MYC	SIRPA	ULBP3
BCL2L1	CD276	GZMH	IL33	NECTIN2	STAT1	VEGFA
BCL6	CD28	GZMK	IL4	NECTIN3	TAP1	VSIR
BID	CD40	GZMM	IL6	NLN	TAP2	VTCN1
BIRC2	CD47	HAVCR2	JAK1	NLRC5	TAPBP	LILRB2
BIRC3	CD48	HHLA2	JAK2	NOS1	TAPBPL	SOCS1
BIRC5	CD70	HLA-A	JAK3	NOS2	TDO2	TNFRSF12A
BIRC6	CD74	HLA-B	KIR2DL1	NOS3	TGFB1	TNFSF10
BIRC7	CD80	HLA-C	KIR2DL3	NRAS	TGFB2	
BIRC8	CD86	HLA-DMA	KIR2DL4	NRDC	TGFB3	
BTLA	CD96	HLA-DMB	KIR3DL1	NT5E	THOP1	
BTN1A1	CEACAM1	HLA-DOA	KIR3DL2	PDCD1	TIGIT	
BTN2A2	CFLAR	HLA-DOB	KIR3DL3	PDCD1LG2	TMIGD2	
BTN3A1	CIITA	HLA-DPA1	KLRB1	PDIA3	TNF	
BTNL2	CRTAM	HLA-DPB1	KLRC1	PRF1	TNFRSF10A	
CADM1	CSF1	HLA-DQA1	KLRC2	PSMB10	TNFRSF10B	
CALR	CSF2	HLA-DQA2	KLRD1	PSMB8	TNFRSF10C	
CANX	CSF3	HLA-DQB1	KLRF1	PSMB9	TNFRSF10D	
CASP1	CTLA4	HLA-DRA	KLRG1	PSME1	TNFRSF14	
CASP10	CTNNB1	HLA-DRB1	KLRK1	PSME2	TNFRSF18	
CASP12	CTSL	HLA-E	KRAS	PSME3	TNFRSF1A	
CASP2	CTSS	HLA-F	LAG3	PTEN	TNFRSF1B	
CASP3	CXCL12	HLA-G	LAP3	PTGS1	TNFRSF21	
CASP4	CXCL17	HRAS	LGALS1	PTGS2	TNFRSF25	
CASP5	CXCL5	ICOS	LGALS3	PVR	TNFRSF4	
CASP6	CXCL8	ICOSLG	LGALS9	PVRIG	TNFRSF6B	
CASP7	CYCS	IDO1	LGMN	RAET1E	TNFRSF9	
CASP8	DFFA	IDO2	LILRB1	RAET1G	TNFSF12	
