# Supplementary material for: Immunogenomic pan-cancer landscape reveals immune escape mechanisms and immunoediting histories
Source: Sci Rep. 2021 Aug 3;11:15713. doi: 10.1038/s41598-021-95287-x (PMC8333422; doi:10.1038/s41598-021-95287-x)
Supplement: Supplementary file 12 — Supplementary Information. [file 41598_2021_95287_MOESM12_ESM.pdf]

Supplementary Information

Immunogenomic Pan-cancer Landscape Reveals Immune Escape Mechanisms and Immunoediting Histories

Shinichi Mizuno[1,†], Rui Yamaguchi[2,†], Takanori Hasegawa[3,†], Shuto Hayashi[2,†], Masashi Fujita[4,†], Fan Zhang[5,†], Youngil Koh[6], Su-Yeon Lee[7], Sung-Soo Yoon[6], Eigo Shimizu[2], Mitsuhiro Komura[2], Akihiro Fujimoto[4], Momoko Nagai[8], Mamoru Kato[8], Han Liang[9], Satoru Miyano[2,3], Zemin Zhang[5,*], Hidewaki Nakagawa[4,*], and Seiya Imoto[2,3,*,10]

[1]Center for Advanced Medical Innovation, Kyushu University, Fukuoka, Japan
[2]Human Genome Center, The Institute of Medical Science, The University of Tokyo, Tokyo, Japan
[3]Health Intelligence Center, The Institute of Medical Science, The University of Tokyo, Tokyo, Japan
[4]Laboratory for Cancer Genomics, RIKEN Center for Integrative Medical Sciences, Yokohama, Japan
[5]BIOPIC and College of Life Sciences, Academy for Advanced Interdisciplinary Studies, Beijing Advanced Innovation Centre for Genomics, Peking University, Beijing, China
[6]Department of Internal Medicine, Seoul National University Hospital, Seoul, Korea
[7]Samsung SDS, Seoul, Korea
[8]National Cancer Research Center, Tokyo, Japan
[9]Department of Bioinformatics and Computational Biology, The University of Texas MD Anderson Cancer Center, Houston, TX, USA
[10]Lead contact
†Equally contributed
*Correspondence: imoto@ims.u-tokyo.ac.jp, hidewaki@riken.jp or zemin@pku.edu.cn

Running title: Immuno-genomic Pan-cancer Landscape

Keywords: Immunogenomic profiling, Pan-cancer analysis, whole genomes, immune escape, immunoediting history

Supplementary Table 1: List of analyzed immune-related genes.

Supplementary Figure 1: Distributions of the determined HLA types from 2,800~ whole genome sequence data. The HLA genotypes were determined by the whole genome sequencing data using our pipeline ALPHARD; its preciseness was 98.6% for class I and 98.2% for class II genes (Hayashi et al., 2018). (a) HLA-A (b) HLA-B (c) HLA-C (d) HLA-DPA1 (e) HLA-DPB1 (f) HLA-DQA1 (g) HLA-DQB1 (h) HLA-DRB1.

Supplementary Figure 2: Somatic mutations in representative immune genes. (a) Identified somatic mutations in *HLA* (class I and class II) and *B2M* genes. The hot spot mutation of *HLA*s indicated by arrow were validated by Sanger sequencing. (b) Allele specific expression of *HLA-A*, *B* and *C* genes. Allelic imbalance in HLA genes in tumor samples could be observed suggesting necessity of investigation of the association with immune escape. (c) Overexpression of 10 immune-related genes and its significant association with SVs in each tumor type. Red and blue dots represent tumor samples with and without SVs, respectively. checkpoint inhibitor.

Supplementary Figure 3: Selective copy number gain and structural variant can explain RNA overexpression. Correlations between occurrence of structural variant and selective copy number gain were observed in *MARCH9* and *SEC61G*. Some samples who have overexpression of these genes without SV have selective copy number gain; selective copy number gain could partly explain molecular mechanism of this overexpression. However, some of other samples with overexpression have neither SV nor selective copy number gain. Further mechanisms might exist.

Supplementary Figure 4: Statistical significance of selective copy number changes. The color of each element represents the score of the statistical test, defined by –sign(t-statistic)*log10(p-value). The function sign(x) takes +1 if x is positive, otherwise -1. Other than the cluster shown in Figure 1c, we observed two interesting clusters, which is located in the right of the heatmap including

TNFRSF6B, *BIRC7*, *CD40*, *BCL2L1*, *EBAG9*, *CCL28*, *SIRPA* and *KRAS*. The heatmap was generated by the R software (R 3.4.0 (April, 2017) (R Core Team, 2017)).

Supplementary Figure 5: Distributions of estimated neoantigen numbers for each tumor type. (a) Neoantigens from SNVs (Class I), (b) Neoantigens from SNVs (Class II) and (c) Neoantigens from indels (ClassI). Neo-antigen predictions in a sample were conducted by using nonsynonymous SNVs and indels and the HLA types of the sample. Binding affinities (IC_{50}) were predicted using netMHCpan3.0 (Nielsen and Andreatta, 2016) for HLA class I and netMHCIIpan3.1 (Andreatta et al., 2015) for HLA class II. Neoantigens were counted for each patient by considering that mutant peptides with IC_{50} values of less than 500 as neoantigens. The samples indicated by red dots are MSI-positive samples.

Supplementary Figure 6: Analysis of infiltrated cells and their predicted activities. Using the results of CIBERSORT and the expression of CD45 for each sample, we estimated the activity of infiltrated immune cells, including CD8+ T-cells, CD4+ T-cells, NK-cells, M2 macrophages, B-cells, etc. An example of a scatter plot (*x*-axis and *y*-axis indicate the predicted activity of CD8+ T-cells and M2 macrophages, respectively) is shown at the bottom, where a circle represents a sample. The dashed red lines are the average values of x and y axes. Even in a tumor type, we could observe diversity of infiltrated immune cells and their activities.

Supplementary Figure 7: Analysis of infiltrated immune cells. (a) Flow cytometry-like plots of the activities of M2 macrophages (y-axis) as immune suppressive cells and CD8+ T-cells (x-axis) as immune effector cells across tumors. The dashed red lines are the average values of x and y axes. (b) The definition of the four regions, R1, R2, R3, and R4. (c) The proportions of donors in each of four areas (R1, R2, R3, and R4). (d) The results of GSEA of Kidney, Lung, Liver and Skin-Melanoma for EMT gene set in the comparison of ‘R2 versus R3’.

Supplementary Figure 8: Comparison of activated NK-cell components of samples in R2 (CD8+/M2+) and R3 (CD8+/M2−) regions. In Supplementary Figure 8 c, GSEA analysis showed different enrichment patterns in EMT across tumor types. It is indicated that those activated NK-cell components tend to be more accumulated in R3 than R2 for kidney and lung cancers, while those tendencies are not clear in liver and skin cancers.

Supplementary Figure 9: Analysis of T cell receptor (TCR). (a) RNA expressions of V genes in TCR alpha. (b) The diversity of TCR repertoire (alpha chain) with inverse Simpson index. (c) The relationships between TCR expression and the diversity of TCR repertoire (d) in lung, kidney, and melanoma (e) and the relationships between CD8A expressions and TCR repertoire diversity. It is observed that kidney cancer has a different tendency showing low TCR diversity even for samples whose sequence reads of TCR sufficiently obtained.

Supplementary Figure 10: Evaluation of the computation of infiltrated immune cells. CIBERSORT deconvolution for the comparison between microarray data and RNA-Seq data using 166 TCGA LAML-US samples. Pearson's correlation coefficients were used to measure concordance. We observed a fine correlation between the immune cell compositions from microarray data and RNA-Seq data using CIBERSORT.
